# Cross‐stage single‐cell and spatial metabolome analyses reveal periderm specialization and tanshinone biosynthesis in *Salvia miltiorrhiza* roots

**DOI:** 10.1111/nph.71285

**Published:** 2026-05-21

**Authors:** Licheng Liu, Lianchong Gao, Zhi‐Ying Wang, Yan Liu, Huifeng Ju, Huana Lin, Zheng Shi, Wen‐Juan Cai, Xue Li, Yan‐Bo Huang, Yu Kong, Jing‐Jing Xu, Hong‐Peng Chen, Xin Fang, Juan Guo, Yong‐Hong Hu, Ke Chen, Xiao‐Ya Chen, Jie Hao, Lei Yang

**Affiliations:** ^1^ Shanghai Key Laboratory of Plant Functional Genomics and Resources Shanghai Chenshan Botanical Garden, and Chenshan Science Research Center, CAS Center for Excellence in Molecular Plant Sciences (CEMPS), Chinese Academy of Sciences (CAS) Shanghai 201602 China; ^2^ University of Chinese Academy of Sciences Shanghai 200032 China; ^3^ Shanghai Center for Systems Biomedicine, Key Laboratory of Systems Biomedicine (Ministry of Education), Shanghai JiaoTong University Shanghai 200240 China; ^4^ School of Traditional Chinese Medicine Guangdong Pharmaceutical University Guangzhou 510006 China; ^5^ Key Laboratory of Phytochemistry and Natural Medicines, Chinese Academy of Sciences Kunming Institute of Botany Kunming 650201 China; ^6^ State Key Laboratory of Plant Trait Design, CEMPS Shanghai Institute of Plant Physiology and Ecology, Chinese Academy of Sciences Shanghai 200032 China; ^7^ State Key Laboratory for Quality Ensurance and Sustainable Use of Dao‐Di Herbs Beijing 100700 China

**Keywords:** diterpene, perennial herb root, *Salvia miltiorrhiza*, single‐cell RNA sequencing, tanshinone biosynthesis

## Abstract

- Perennial herbs develop long‐lived roots that undergo repeated cycles of secondary growth, during which the periderm functions as a protective barrier and a major site of specialized metabolite accumulation. However, the cellular programs that coordinate periderm differentiation and secondary metabolism in perennial roots remain poorly understood.
- Single‐cell RNA sequencing, together with spatial metabolome analyses, was employed to resolve cell‐type composition and transcriptional dynamics in *Salvia miltiorrhiza* roots at different developmental stages.
- Based on Batch Integration via Precision Annotation and Consistency Testing‐integrated single‐cell data, the developmental trajectory from epidermal to peridermal lineages was reconstructed, revealing distinct transcriptional programs associated with diterpenoid biosynthesis. Spatial metabolome analyses confirmed that tanshinones were predominantly accumulated in cork cells of mature roots. Integrative analysis further indicated that SmCYP76AK5 participates in tanshinone biosynthesis.
- This cellular framework enables the prediction of metabolic activities in specific cell types and facilitates the inference of developmental differences between young and mature roots. It also provides a basis for integrating single‐cell data into models of secondary metabolism in perennial plants.

## Graphical abstract

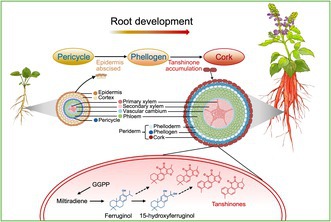

## 概括

- 多年生草本植物的根系寿命较长，其发育经历次生生长，在这一过程中，周皮既是重要的保护屏障，也是特殊化合物积累的主要部位。然而，多年生根的周皮分化与次生代谢之间如何在细胞层面相互协调，目前仍不清楚。
- 本研究结合单细胞转录组测序和空间代谢组分析，对不同发育阶段丹参(*Salvia miltiorrhiza*)根中的细胞类型组成及转录动态进行了系统解析。
- 基于BIPACT整合的单细胞数据，构建了由表皮细胞谱系向周皮细胞谱系转变的发育轨迹，并发现了与二萜类生物合成相关的差异性转录表达。空间代谢组结果进一步显示，丹参酮类成分主要积累于成熟根的木栓层细胞中。通过多组学整合分析，研究还发现SmCYP76AK5参与了丹参酮类化合物的生物合成。
- 本研究建立了一个细胞分辨率的分析框架，可用于预测特定细胞类型中的代谢活性，并有助于理解幼年和成熟根之间的发育差异，同时也为多年生药用植物次生代谢研究中整合单细胞数据提供了范例。

## Introduction

Perennial species undergo repeated cycles of growth and secondary development, resulting in storage roots with structural complexity and metabolic specialization absent from annuals (Gonzalez‐Paleo *et al*., 2024). Perennial, herbaceous plants develop extensive root systems that enhance belowground biomass and help reduce soil loss (Ciotir *et al*., 2019). The maturation of belowground storage and clonal organs is a slow, long‐term process that poses challenges for conventional experimental analyses (Martínková *et al*., 2025).

During secondary growth, the periderm acts as a durable armor in both woody and herbaceous plants, replacing the primary protective tissues such as the epidermis and endodermis. It safeguards the plant from pathogen invasion and abiotic stresses, while sealing the outer surface during wounding and organ abscission (Serra *et al*., 2022). The periderm, initiated by phellogen derived from the pericycle, ultimately replaces the epidermis as the outer protective barrier (Wunderling *et al*., 2018). Although periderm formation has been extensively studied in annual models such as Arabidopsis (Miller *et al*., 2025), the cellular mechanisms governing periderm differentiation and secondary metabolite accumulation in perennial roots remain poorly understood.

*Salvia miltiorrhiza* (Danshen), a perennial medicinal herb, provides an excellent model for studying root periderm specialization and secondary metabolism. Its roots undergo continuous remodeling of the periderm over multiple years, serving both as a protective barrier and as the major site of tanshinone accumulation (Xu & Song, 2017; Tong *et al*., 2022; Xia *et al*., 2023). Among the major active constituents, abietane‐type diterpenoids known as tanshinones accumulate predominantly in the root periderm, highlighting the close relationship between periderm development and secondary metabolism in perennial roots (Xu *et al*., 2015). Despite recent advances in genome sequencing and biosynthetic pathway elucidation (Ma *et al*., 2021; Tong *et al*., 2022; Hu *et al*., 2023), the cellular origin and the relationship between the regulatory basis of periderm formation and tanshinone biosynthesis remain largely unexplored.

Single‐cell RNA sequencing (scRNA‐seq) enables high‐resolution characterization of transcriptional heterogeneity, providing insights into cell identity and developmental trajectories (Satterlee *et al*., 2020; Seyfferth *et al*., 2021; Shaw *et al*., 2021; Denyer & Timmermans, 2022; Xue *et al*., 2025). This approach provides fundamental insights into organ development, cell differentiation, and cell division (Liu *et al*., 2021; Y. Wang *et al*., 2021; Xu *et al*., 2022; Tung *et al*., 2023). In plants, scRNA‐seq has resolved cell‐type‐specific biosynthetic specialization, such as the identification of catechin‐modifying glycosyltransferases in *Camellia sinensis* (Wang *et al*., 2022), and alkaloid‐pathway compartmentalization in *Catharanthus roseus* (Li *et al*., 2023; Sun *et al*., 2023). These advances underscore that secondary metabolism is often spatially organized among distinct cell types. A single‐cell framework is therefore required to uncover the unique developmental and metabolic features of perennial roots such as those of *S. miltiorrhiza*.

To overcome the limitations of established methods, we performed scRNA‐seq analyses of *S. miltiorrhiza* roots at two developmental stages, representing young (2‐month‐old plants) and mature (2‐yr‐old plants), to capture the developmental trajectory of periderm formation and diterpenoid biosynthesis. We developed BIPACT (Batch Integration via Precision Annotation and Consistency Testing), a deep learning algorithm designed for precise dataset integration and consistent cell‐type identification. Using this integrative framework, we delineated tanshinone biosynthesis during periderm specialization and uncovered key enzyme genes involved in this pathway. Specifically, we characterized a cytochrome P450 (SmCYP76AK5) that catalyzes the C‐15 hydroxylation of ferruginol. Together, these findings establish a cellular framework for understanding periderm differentiation and reveal the cell‐type‐specific basis of secondary metabolite biosynthesis in a perennial root system.

## Materials and Methods

### Plant materials

Root tissues from *Salvia miltiorrhiza* Bunge (sourced and identified from the Shanghai Chenshan Botanical Garden Nursery) at growth stages of 15 d, 1 month, 2 months, 3 months, and 2 yr were collected.

### Chemicals

Ferruginol and 15‐hydroxyferruginol were purchased from BioBioPha (Kunming, China) and Catch Bioscience & Technology Co., Ltd (Suzhou, China), respectively.

### Extraction of the root tissues in *S. miltiorrhiza*

The roots were promptly cleaned, cut into small segments, and placed into labeled centrifuge tubes. The samples were flash‐frozen in liquid nitrogen for 3 min and then lyophilized under vacuum for 24 h. The dried samples were ground into fine powder using a mortar, and 0.05 g of each sample was extracted with 1 ml of HPLC‐grade methanol via ultrasonication for 1 h. During ultrasonic extraction, samples were continuously cooled with ice, and the temperature of the ultrasonic bath was monitored in real time and maintained below 20°C to minimize potential metabolite degradation. The extracts were centrifuged at 13 523 ***g*** for 10 min at room temperature, and the supernatant was filtered through a 0.22 μm membrane for subsequent LC‐MS analysis.

### LC‐MS analysis

LC‐MS analysis was performed in an untargeted full‐scan mode using a Thermo Fisher Scientific instrument (Waltham, MA, USA). Chromatographic separation was achieved on a Waters ACQUITY C18 column (2.1 × 100 mm, 1.7 μm), with the column temperature maintained at 40°C and a flow rate of 0.4 ml min^−1^. The mobile phase consisted of 0.1% formic acid in water (A) and 0.1% formic acid in acetonitrile (B), with an injection volume of 2 μl. Mass spectra were acquired in both positive and negative ion modes, scanning an *m/z* range of 50–450. Total ion chromatograms were generated from the full‐scan data to visualize global metabolite profiles. For targeted analysis of specific compounds, extracted ion chromatograms (EICs) were generated based on their exact *m/z* values. Authentic standards of ferruginol (1), cryptotanshinone (2), dihydrotanshinone I (3), and tanshinone IIA (4) were analyzed under identical conditions for validation. All data were collected and analyzed using the Xcalibur 2.1 software (Thermo Fisher, USA).

### Microscopy

Roots from 2‐month‐old and 2‐yr‐old *S. miltiorrhiza* plants were used for microscopy analysis. Root segments were sampled from the middle region of the roots (root mid‐zone). Specifically, segments located 4–6 cm from the root tip were collected from 2‐month‐old plants, whereas segments located 10–12 cm from the root tip were collected from 2‐yr‐old plants. Root samples were manually dissected and embedded in 4% (w/v) molten agarose. Transverse sections (240 μm thickness) were obtained using a vibrating microtome (Leica VT1200S; Leica Microsystems, Wetzlar, Germany). Sections were stained with SCRI Renaissance 2200 (SR2200) as previously described (Musielak *et al*., 2015). Microscopic observation was performed using an Olympus SpinSR microscope. SR2200 fluorescence was excited with a 405‐nm laser, and emission was detected between 417 and 477 nm (EX405/EM417‐477). Autofluorescence signals were acquired using a 488‐nm laser, with emission collected between 500 and 550 nm (EX488/EM500‐550).

### AP‐MALDI MSI

Samples were collected 4–6 cm from the root tip in 2‐month‐old plants and 10–12 cm from the root tip in 2‐yr‐old *S. miltiorrhiza* plants. The root regions were cleaned, and a 1.0‐ to 1.5‐cm segment was excised and immediately frozen in liquid nitrogen for 20 s, followed by storage at −80°C.

The tissue was equilibrated in a cryostat for 3 h, embedded in carboxymethylcellulose, and sectioned into 10‐μm slices using a cryostat microtome. The sections were transferred onto precooled indium tin oxide conductive slides using a chilled brush and dried for 20 min. A matrix solution of α‐cyano‐4‐hydroxycinnamic acid at a concentration of 10 mg ml^−1^ in chloroform : methanol (3 : 1, v/v) containing 0.1% trifluoroacetic acid was applied using a robotic aerosol sprayer (SunCollect; SunChrom, Friedrichsdorf, Germany). The nozzle velocity was set to 1000 mm min^−1^, and 18 layers were deposited with a gradient flow rate ranging from 10 to 60 μl min^−1^. A Thermo Q Exactive Plus mass spectrometer (Thermo Fisher Scientific) coupled with an AP‐MALDI ion source was used for detection. The spatial resolution was set to 17‐μm pixel size. The laser energy was set to 15%, and data were acquired in positive ion mode with a full‐scan range of 120–1000 *m/z* at a resolution of 35 000. The maximum ion injection time was 100 ms, automatic gain control was set to 1 × 10^6^, the isolation window was set to 2 *m/z*, the capillary temperature was 350°C, and the S‐lens RF level was 50%.

In the mass spectrometry imaging (MSI) experiment, the atmospheric pressure matrix‐assisted laser desorption/ionization (AP‐MALDI) ion source scanned the sample line‐by‐line, and the acquired spectra were saved as RAW files. The MSI data were processed and visualized using a proprietary software platform (http://www.biodeep.cn). The RAW files were first converted into the open source mzML format. Peak detection and quantification were performed, and the TrIQ algorithm was applied to distinguish background noise and correct signals. Molecules within the *m/z* range of 100–1000 were identified, and the data were subjected to dimensionality reduction and background subtraction before downstream analysis.

To optimize AP‐MALDI‐MSI, standard compounds of tanshinones were prepared as references and for optimizing detection conditions for similar compounds.

### Protoplast isolation

Root tissue sampling was consistent with that for microscopy observation. Root segments were collected 4–6 cm from the root tip in the 2‐month‐old plants and 10–12 cm from the root tip in the 2‐yr‐old *S. miltiorrhiza* plant. For protoplast isolation, segments of *c*. 2.0 cm in length were excised from these regions. An enzyme solution was prepared by mixing Cellulase R‐10, Macrozyme R‐10, Mannitol, KCl, and MES, heating at 55°C for 3–4 min, cooling, and then adding CaCl_2_ solution and BSA powder. The root samples were sliced into 60‐μm sections, incubated in enzyme solution in the dark for 40 min at room temperature, and transferred into RNase‐free 10 ml centrifuge tubes containing the enzyme solution. The samples were enzymatically digested at 26–27°C for 8 h, with gentle agitation every hour to facilitate cell dissociation. The dissociated solution was filtered through a 40‐μm sterile mesh and centrifuged at 150 ***g*** for 6 min at 4°C. The supernatant was discarded, and the pellet was washed twice with ice‐cold wash solution, followed by centrifugation at 150 ***g*** for 5 min. The protoplasts were resuspended in 1 ml of 8% mannitol, mixed with 4% trypan blue stain (1 : 10 ratio), and counted using a hemocytometer under a microscope. Viable protoplasts remained unstained, while ruptured or dead cells were stained blue.

### ScRNA sequencing

Protoplasts were counted under a microscope using a hemocytometer, ensuring a concentration of 600–2000 cells μl^−1^ and viability exceeding 80%. The Chromium Single Cell 30 V3 Reagent Kit (10x Genomics, Pleasanton, CA, USA) was used to prepare single‐cell microreactors. Gel Beads with barcodes, oil, and enzyme‐containing reagents were mixed with the cell suspension and loaded into the instrument. The microfluidic ‘double‐cross’ channels generated oil–water emulsion droplets, encapsulating single cells and barcoded Gel Beads. The Gel Beads dissolved, releasing barcoded capture sequences that bound to mRNA released from lysed protoplasts, tagging each transcript with a unique molecular identifier (UMI). Reverse transcription was performed in a PCR machine for 25 min. After breaking the emulsion, the cDNA was pooled, amplified via PCR, and quantified using Qubit. Sequencing libraries were constructed and sequenced on the Illumina HiSeq platform using PE150 mode.

### ScRNA‐seq data analysis

Sequencing reads were aligned to the reference genome of *S. miltiorrhiza* (GCF_028751815.1_IMPLAD_Smil_shh) using Cell Ranger for quality control (QC) UMI correction, and counting. A feature‐barcode matrix was generated (genes as features and cell barcodes as columns/identifiers). We filtered out low‐quality cells based on standard QC criteria, including elevated mitochondrial/chloroplast transcript fractions and predicted doublets, retaining high‐confidence viable cells for downstream analysis. The seurat package in R was used to create an object from the filtered barcodes, genes, and matrix files. Data quality control involved removing cells with fewer than 200 expressed genes and genes detected in fewer than 20 cells. The NormalizeData algorithm was applied for expression normalization. To mitigate batch effects, a novel BIPACT algorithm was developed and compared with scVI, BERMUDA, Harmony, CCA, scArches, rPCA, and BBKNN. Cells were clustered in the principal component analysis (PCA) space using a graph‐based approach, and uniform manifold approximation and projection (UMAP) was used for visualization. The FindAllMarkers function identified differentially expressed marker genes, which were analyzed for pathways (Kyoto Encyclopedia of Genes and Genomes (KEGG)) and biological processes (Gene Ontology (GO)). Cell type annotation was performed using homologous marker genes from Arabidopsis and the ScType platform. RNA velocity and pseudotime analyses were performed to infer directional developmental dynamics, while MetaNeighbor was used to assess cross‐sample cell‐type reproducibility.

### Phylogeny of candidate CYPs

All cytochrome P450 (CYP) genes from the target cell cluster were used for phylogenetic analysis. Amino acid sequences were aligned using MUSCLE v.3.8.31 (http://www.drive5.com/muscle). Phylogenetic trees were constructed with RAxML‐NG v.0.9.0 (https://github.com/amkozlov/raxml‐ng), using the LG + I + G substitution model, which was determined as the best‐fit model by ProtTest v.3.4.2 (https://github.com/ddarriba/prottest3). Functionally characterized CYPs from *S. miltiorrhiza* were included for reference. Bootstrap analysis was performed with 1000 replicates to assess branch support.

### Gene isolation and plasmid construction

Total RNA was isolated from *S. miltiorrhiza* and subsequently reverse‐transcribed into cDNA using the HiScript III 1st Strand cDNA Synthesis Kit (+gDNA wiper) (Vazyme, Nanjing, China). The open reading frames (ORFs) of all genes involved in the diterpenoid pathway, *SmGGPPS*, *SmCPS*, *SmKSL*, *SmCYP76AH1*, *SmCPR*, and *SmCYP76AK5*, were amplified and cloned into two expression vectors using the ClonExpress II One Step Cloning Kit (Vazyme). For yeast expression, the ORF was inserted into the pAg423Gal‐ccdb vector using *SpeI* and *XhoI* restriction sites, while for *Nicotiana benthamiana* expression, it was cloned into the pEAQ‐HT vector via *AgeI* and *XhoI*. Primer sequences used for PCR amplification are listed in the Supporting Information Table S1. Recombinant plasmids were introduced into *Escherichia coli* DH10B cells, and transformants were selected on Luria–Bertani (LB) agar plates containing appropriate antibiotics. Positive colonies were identified by colony PCR using the primers also in the Table S1 and verified by Sanger sequencing after plasmid extraction from overnight cultures.

### Subcellular localization of CYP76AK5 in *N. benthamiana*

The full‐length *CYP76AK5* coding sequence was inserted into pEAQ and fused to GFP to generate the *35S::CYP76AK5‐GFP* plasmid. The construct was transformed into *Agrobacterium tumefaciens* strain GV3101 and transiently expressed in *N. benthamiana* leaves through injection. The fluorescence signal was detected using Leica TSC SP8 STED 3× (Leica) 72 h after injection.

### CRISPR‐Cas9 mutagenesis and plant transformation

For the CRISPR‐Cas9 construction targeting the *SmCYP76AK5* coding region, 2 gRNA target sites were designed by CRISPOR (http://crispor.gi.ucsc.edu). To produce the gRNAs, a PCR was carried out with primers containing both the gRNA sequences (Table S1) and cloned into the plasmid pTargetF with AtU6pro (Addgene plasmid 62226). The Level 1 vectors pICSL11059‐2x35S::proNPTII (Addgene plasmid 68263), pICH47742‐RPS5Apro::Cas9 (Addgene plasmid 48001), pICH47761‐AtUBi10pro::dsRED (Addgene plasmid 48003), and pICH41780 (Addgene plasmid 48019) were then assembled into the binary level 2 vector pAGM4723 (Addgene plasmid 48015), using BbsI and BsaI enzymes to obtain the final binary plasmid. The CRISPR‐assembled construct was transformed into *A. tumefaciens* K599 and introduced into *S. miltiorrhiza* leaf explants, and hairy roots were induced as described previously (Song *et al*., 2022).

### Mutation validation and complementation assay

Target regions were amplified by PCR and sequenced to confirm deletions. *SmCYP76AK5* was introduced into the pK7WG2R vector between the 35S promoter and 35S terminator. The constructs were then used to transform *A. tumefaciens* strain K599 and introduced into the mutant hairy root *ak5‐8*, which were cultured for 3 wk and then harvested.

### RT‐qPCR analysis

Total RNA was extracted from CRISPR‐Cas9 lines and complement lines using RNeasy Plant Kit (Qiagen, Hilden, Germany). cDNA was synthesized with PrimeScript RTase (TaKaRa, Shiga, Japan). Reverse transcription quantitative polymerase chain reaction (RT‐qPCR) was performed on QuantStudio 5 (Thermo Fisher) with SYBR Green Master Mix, using *SmActin* as reference gene. Three biological replicates (independent transgenic lines) were analyzed with three technical replicates each. The 2 ^(−ΔΔ*Ct*)^ method was used for quantification.

### Transient expression in *N. benthamiana*

The recombinant plasmid of pEAQ‐HT‐GGPPS, pEAQ‐HT‐CPS, pEAQ‐HT‐KSL, pEAQ‐HT‐CYP76AH1, pEAQ‐HT‐CPR, and pEAQ‐HT‐CYP76AK5 was transferred to *A. tumefaciens* strain GV3101 (pSoup‐p19) (Weidi Biotechnology, Shanghai, China) and introduced into *N. benthamiana* by agro‐infiltration. Transformed *A. tumefaciens* colonies were identified by PCR, and a positive colony was cultured in 10 ml LB containing 50 μg ml^−1^ rifampicin and 50 mg ml^−1^ kanamycin and grown for 1–2 d at 200 rpm, 28°C. Cells were centrifuged at 1646 ***g*** for 10 min, then resuspended in buffer (10 mM MgCl_2_, 10 mM MES, 150 mM acetosyringone). All transformed *A. tumefaciens* suspensions were normalized to OD_600_ *c*. 1.0 and kept in dark for almost 2–3 h. Co‐infiltrations with empty vector pEAQ‐HT were used as the negative control.

### Heterologous expression in yeast

Recombinant *Saccharomyces cerevisiae* strains harboring the gene *SmCYP76AK5* were first cultured in synthetic dropout (SD) medium containing 2% glucose at 30°C, 220 rpm for 12 h. The cells were then harvested by centrifugation and resuspended in fresh SD medium containing 2% galactose to induce gene expression. Induction was carried out at 30°C with shaking for an additional 24 h. After induction, yeast cultures were centrifuged at 1646 ***g*** for 10 min and extracted with methanol. The detection procedures were performed in accordance with the protocols used in the LC‐MS analysis.

### NMR analysis

^1^H and ^13^C NMR spectra were recorded on a Bruker Avance III 600 MHz spectrometer (Bruker BioSpin, Ettlingen, Germany) at 298 K using CDCl_3_ as the solvent. Chemical shifts were calibrated against residual solvent peaks. Complete spectral data and structural assignments for 15‐hydroxyferruginol are provided here:

^1^H NMR (600 MHz, CD_3_OD) 6.81 (s, 1H), 6.63 (s, 1H), 3.35 (s, 1H), 2.82 (dd, *J* = 18.0, 6.0 Hz, 1H), 2.72 (m, 1H), 2.23 (br. d *J* = 12.0 Hz, 1H), 1.84 (m, 1H), 1.79 (dt, *J* = 12.0, 6.0 Hz, 1H), 1.68 (m, 1H), 1.60 (dt, *J* = 18.0, 6.0 Hz, 1H), 1.59 (s, 3H), 1.55 (s, 3H), 1.48 (br. d *J* = 12.0 Hz, 1H), 1.33 (td, *J* = 12.0, 6.0 Hz, 1H), 1.24 (td, *J* = 12.0, 6.0 Hz, 1H), 1.17 (s, 3H), 0.96 (s, 3H), 0.94 (s, 3H); ^13^C NMR (600 MHz, CD_3_OD) *δ* 152.77 (C), 149.74 (C), 129.88 (C), 125.45 (CH), 124.37 (C), 111.62 (CH), 73.49 (C), 50.68 (CH), 41.52 (CH_2_), 38.76 (CH_2_), 37.31 (C), 33.01 (C), 32.43 (CH_3_), 29.57 (CH_2_), 28.93 (CH_3_), 28.92 (CH_2_), 23.87 (CH_3_), 20.70 (CH_3_), 19.08 (CH_2_), 18.97 (CH_2_).

## Results

### Unveiling cellular heterogeneity of Danshen roots at two developmental stages by scRNA‐seq

**Fig. 1:**
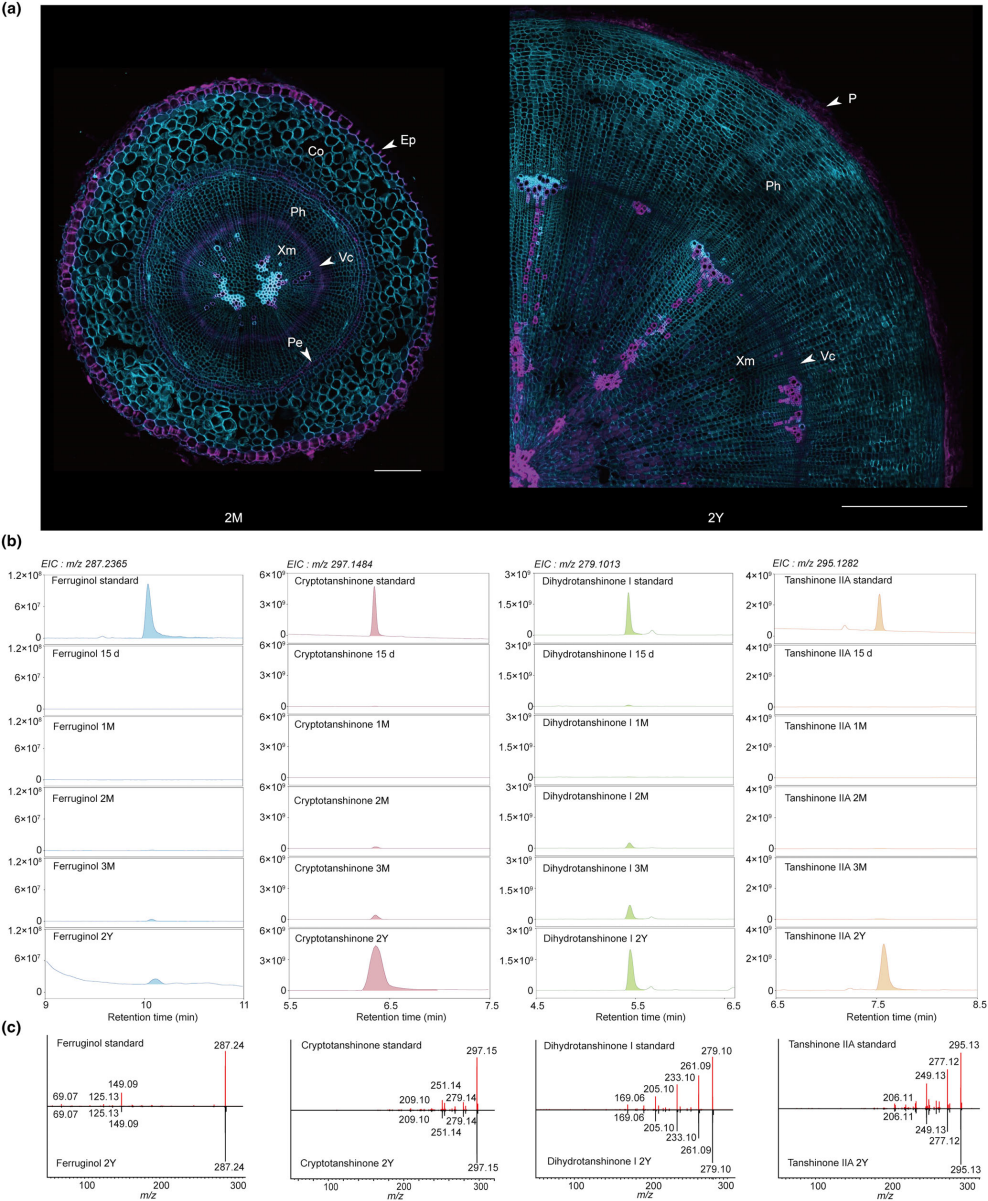
Microscopy, targeted LC‐MS profiling and anatomical characterization of young and mature *Salvia miltiorrhiza* roots. (a) Microscopic images of roots from young (2‐month‐old plants, 2M) and mature (2‐yr‐old plants, 2Y) *S. miltiorrhiza*. Autofluorescence signals were collected using a 488‐nm laser and detected at 500–550 nm (magenta), highlighting the epidermis (Ep), pericycle (Pe), vascular cambium (Vc), periderm (P), and xylem vessels. Cell walls were stained with SCRI Renaissance 2200 (SR2200) and visualized in blue using excitation at 405 nm and emission detection at 417–477 nm, enabling the identification of cortex (Co), phloem (Ph), and xylem (Xm) cells. Bars, (2M) 200 μm; (2Y) 1 mm. (b) Extracted ion chromatograms of major tanshinone compounds in *S. miltiorrhiza* roots at different developmental stages (15‐d‐old, 1‐month‐old, 2‐month‐old, 3‐month‐old and 2‐yr‐old plants), together with authentic standards. Ferruginol (blue), cryptotanshinone (pink), dihydrotanshinone I (green), and tanshinone IIA (yellow) are indicated by color‐coded peak areas. (c) Comparative MS/MS fragmentation patterns of major tanshinone compounds and corresponding standards shown in (b).

*Salvia miltiorrhiza* is a perennial herb with storage roots. Its thickened root, which undergoes secondary growth, is mainly composed of periderm. Comparing root cross sections from 2‐month‐old and 2‐yr‐old plants, the epidermis and cortex were absent in roots from 2‐yr‐old plants, indicating that a well‐developed and mature secondary structure had formed (Fig. 1a). To characterize the spatial accumulations of tanshinones and other abietane‐type diterpenoids during root development, we collected root samples from *S. miltiorrhiza* plants aged from 15 d to 2 yr. Metabolite profiling was conducted using ultra‐high‐performance liquid chromatography coupled with quadrupole orbitrap high‐resolution mass spectrometry (Q Exactive, QE). Targeted LC‐MS analysis revealed clear stage‐dependent differences in tanshinone accumulation during root development (Figs 1b, S1). EICs of major tanshinones, including ferruginol, cryptotanshinone, dihydrotanshinone I, and tanshinone IIA, showed markedly stronger signals in roots from 2‐yr‐old plants compared to the roots of earlier developmental stages, in which only low‐intensity peaks were detected. The identities of these compounds were further confirmed by matching retention times and MS/MS fragmentation patterns with authentic standards (Fig. 1c). Notably, MS/MS spectra acquired from root extracts of 2‐yr‐old plants exhibited high consistency with those of the corresponding standards, providing robust support for compound identification. Together, these results demonstrate a pronounced developmental activation of tanshinone biosynthesis during root maturation.

To further dissect the cellular basis of this tissue‐specific and stage‐dependent metabolic specialization, we employed scRNA‐seq to resolve the transcriptional landscape of root cells at early and later stages, in order to address the cellular heterogeneity that cannot be captured by bulk RNA‐seq. First, we isolated viable protoplasts from the root tissues (Fig. S2). Then, using the 10× Genomics platform for scRNA‐seq, we obtained transcriptomic profiles at single‐cell resolution. The reads were mapped to the reference genome (Pan *et al*., 2023), with 90.5% of reads mapping in samples from 2‐yr‐old plants (2Y) and 89.5% in samples from 2‐month‐old plants (2M), which demonstrated the high quality and reliability of the transcriptome sequencing data. In total, 14 524 single cells were captured from the 2Y, with an average of 1531 genes detected per cell. Meanwhile, from the 2M, 13205 high‐quality cells were obtained, among which a median of 876 genes per cell were identified (Table S2).

**Fig. 2:**
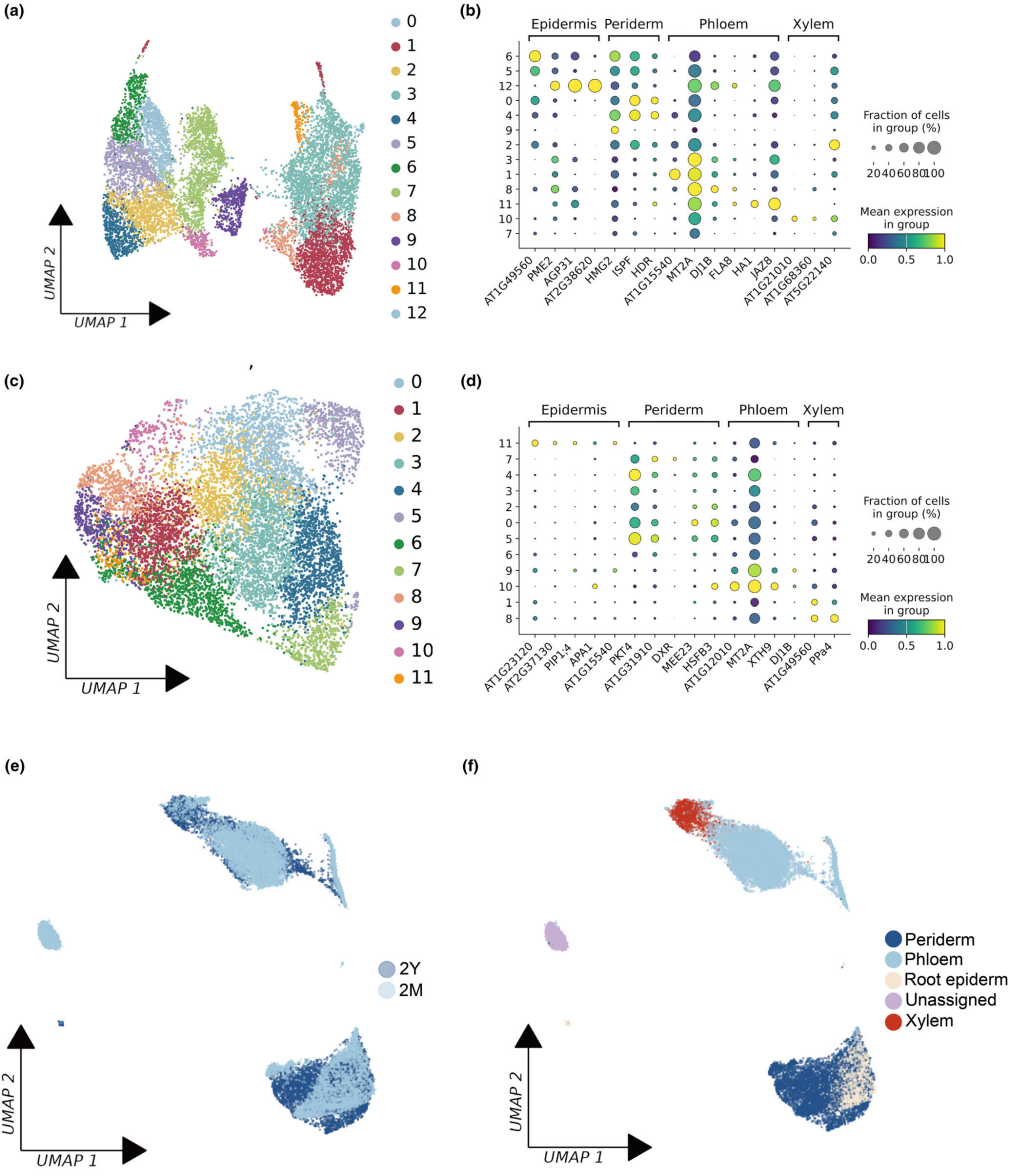
Developmental stage‐resolved single‐cell landscape of *Salvia miltiorrhiza* roots from 2M and 2Y plants. (a, c) Cluster analysis and cell type identification of single‐cell RNA sequencing data from 2M and 2Y samples, respectively. (b, d) Annotations of cell clusters in 2M and 2Y samples by DsAno referring to epidermis, periderm, phloem, and xylem. (e) Integration of 2M and 2Y samples, generated using the BIPACT algorithm. (f) Corresponding atlas annotated with major tissue identities, including epidermis, periderm, phloem, xylem, and unassigned.

Typically, annotation of cell types in plant scRNA‐seq data relies on well‐characterized marker genes. However, the identification of such reliable marker genes in *S. miltiorrhiza* remains elusive, as the limitations of conventional annotation approaches are often insufficient for the specific requirements for annotating single‐cell data in *S. miltiorrhiza* (Fig. S3). Inspired by a synteny‐based workflow (Wu *et al*., 2024), we developed DsAno, a tailored annotation workflow for *S. miltiorrhiza* (DsAno) (Fig. S4; Tables S3, S4). To identify transcriptionally distinct populations, we performed PCA for dimensionality reduction, followed by graph‐based clustering on the reduced space; UMAP was used for visualization of the resulting clusters. As a result, both the 2Y and the 2M root samples were annotated by DsAno, respectively, revealing four common cell types: phloem, xylem, epidermis, and periderm (Fig. 2a–d). Additionally, MetaNeighbor (Crow *et al*., 2018) was used to check the consistency of annotated cell types between the two samples, providing an independent validation of cell‐type reproducibility between 2M and 2Y (Fig. S5).

Next, we developed a semisupervised sample integration algorithm based on deep learning, BIPACT (Fig. S6; Methods S1). This scheme, using previously annotated cell types as reference, along with consistency verification of DsAno and MetaNeighbor, allowed us to accurately identify reliable cell type annotations. Accordingly, we defined a set of high‐confidence shared cell types (anchors) to guide batch integration (Jean‐Baptiste *et al*., 2019). We first benchmarked BIPACT using *Arabidopsis thaliana* root single‐cell datasets to evaluate its performance. BIPACT yielded significantly more distinct and compact cell clustering compared to conventional approaches, including scVI (Lopez *et al*., 2018), BERMUDA (Wang *et al*., 2019), Harmony (Korsunsky *et al*., 2019), CCA (Hardoon *et al*., 2004), scArches (Lotfollahi *et al*., 2021), rPCA (Partridge & Jabri, 2000), BBKNN (Polański *et al*., 2019), and the uncorrected data (Fig. S7). Subsequently, we applied BIPACT to our dataset. Consistent with the benchmark results, BIPACT outperformed alternative strategies, demonstrating superior efficacy in removing technical batch effects while preserving stage‐dependent transcriptional differences within each cell type (Fig. S8). Notably, in the BIPACT results, we found that periderm and epidermis appeared closely adjacent in latent space (Fig. 2e,f), a pattern also observed in CCA and scVI results, but being more distinctly resolved by BIPACT (Fig. S8).

### Periderm differentiation and cellular basis of tanshinone biosynthesis

**Fig. 3:**
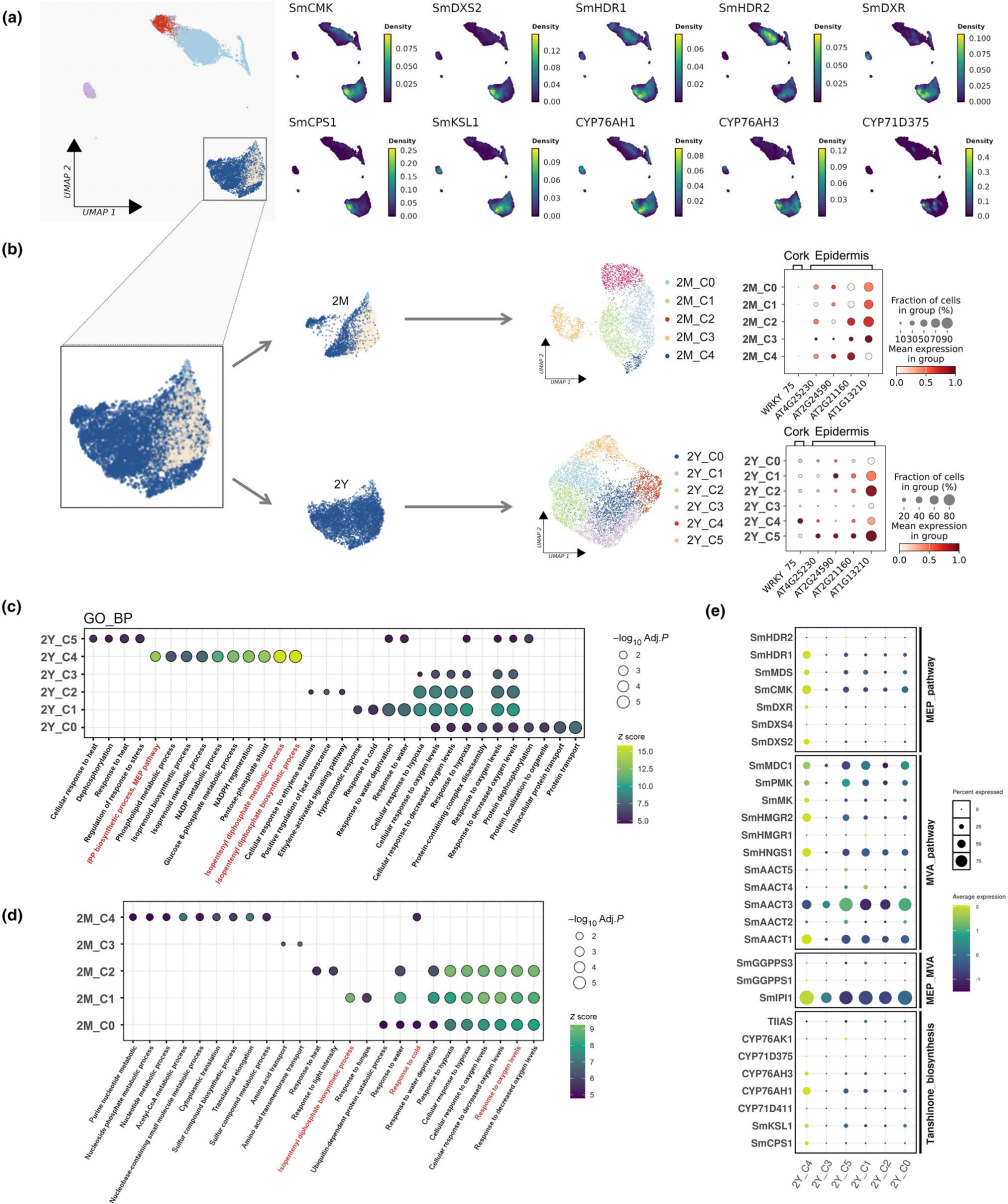
Analysis of periderm‐related cells in *Salvia miltiorrhiza* roots from 2M and 2Y plants. (a) Featureplot of the genes involved in the 2‐C‐methyl‐D‐erythritol 4‐phosphate pathway (*SmCPS1*, *SmKSL*, *SmCMK*, *SmDXR2*, *SmHDR1*, *SmHDR2*, and *SmDXR*) and the cytochrome P450 family (*CYP76AH1*, *CYP76AH3*, and *CYP71D375*) in the integrated single‐cell data of 2M and 2Y. (b) Schematic representation of reclustered cell populations from each sample, with validation of marker genes for cork and epidermal cells. Subclusters 2M_C3 and 2Y_C5 correspond to epidermal cells, whereas 2Y_C4 corresponds to cork cells. (c, d) Gene Ontology enrichment analysis of the pathways related to terpenoid biosynthesis in the 2Y sample (marked in red), and in the 2M sample, with the focus on the processes related to environmental response (marked in red). (e) Bubble plot of the expression of the known tanshinone biosynthesis pathway genes in the 2Y group.

The biosynthesis of tanshinones involves three major stages. First, the universal diterpenoid precursors isopentenyl diphosphate (IPP) and dimethylallyl pyrophosphate are synthesized via the 2‐C‐methyl‐D‐erythritol 4‐phosphate (MEP) pathway in plastids. These C5 basic units are condensed to geranylgeranyl diphosphate (GGPP) by prenyl transferase, followed by cyclization by copalyl diphosphate synthase (SmCPS) and kaurene synthase‐like (SmKSL), also in plastids, to produce the miltiradiene backbone (Gao *et al*., 2009; Hu *et al*., 2023). Finally, miltiradiene undergoes oxidative modifications in the cytoplasm by oxidoreductases, such as CYP monooxygenases (Guo *et al*., 2013; Ma *et al*., 2021) and 2‐oxoglutarate‐dependent dioxygenases (Song *et al*., 2022), to generate diverse abietane‐type diterpenoids, including tanshinones (Fig. S9). To investigate the relationship between tanshinone biosynthesis and root development, we analyzed biosynthetic gene expression in young and mature roots. After BIPACT integration, the transcripts of tanshinone biosynthetic genes were predominantly enriched in the cells annotated as periderm or epidermis, including *SmCPS1*, *SmKSL*, *SmCMK*, *SmDXR2*, *SmHDR1*, *SmHDR2*, *SmDXR*, *CYP76AH1*, *CYP76AH3*, and *CYP71D375*. Among them, *SmHDR1* and *SmHDR2*, encoding 4‐hydroxy‐3‐methylbut‐2‐enyl diphosphate reductase of the MEP pathway, also showed detectable expression in the phloem (Fig. 3a; Table S5).

Given the strong periderm‐associated expression of these genes, we subsequently extracted and separately processed the cell clusters adjacent to periderm. We further performed individual clustering analyses on the cells from 2‐month‐old (2M) and 2‐yr‐old (2Y) samples. The 2M group was divided into five subclusters (2M_C0, 2M_C1, 2M_C2, 2M_C3, 2M_C4), while the 2Y group yielded six subclusters (2Y_C0, 2Y_C1, 2Y_C2, 2Y_C3, 2Y_C4, 2Y_C5) (Figs 3b, S10). At the same time, we adopted conserved marker genes previously identified as cork and epidermis in Arabidopsis for the identification of both 2M and 2Y samples. We found that cork cells were present exclusively in the 2Y subcluster 2Y_C4 and were nearly absent in 2M and that the 2M_C3 and 2Y_C5 subclusters were annotated as epidermis (Fig. 3b–d). To define these subclusters, biological process category based on GO enrichment analysis was conducted, which revealed that the 2Y sample was significantly enriched in biological processes associated with secondary metabolism, including the MEP pathway and the IPP metabolism, whereas the 2M sample was predominantly enriched in processes related to environmental response (Fig. 3c,d). Moreover, in the 2Y sample, the tanshinone biosynthetic genes (*SmCPS1*, *SmKSL*, *CYP76AH1*, *CYP76AH3*, and *CYP71D375*) were highly expressed in subcluster 2Y_C4, as revealed by both visualization and quantitative analysis (Fig. 3e; Table S6).

### Pseudotime analysis unravels the developmental trajectory of periderm

**Fig. 4:**
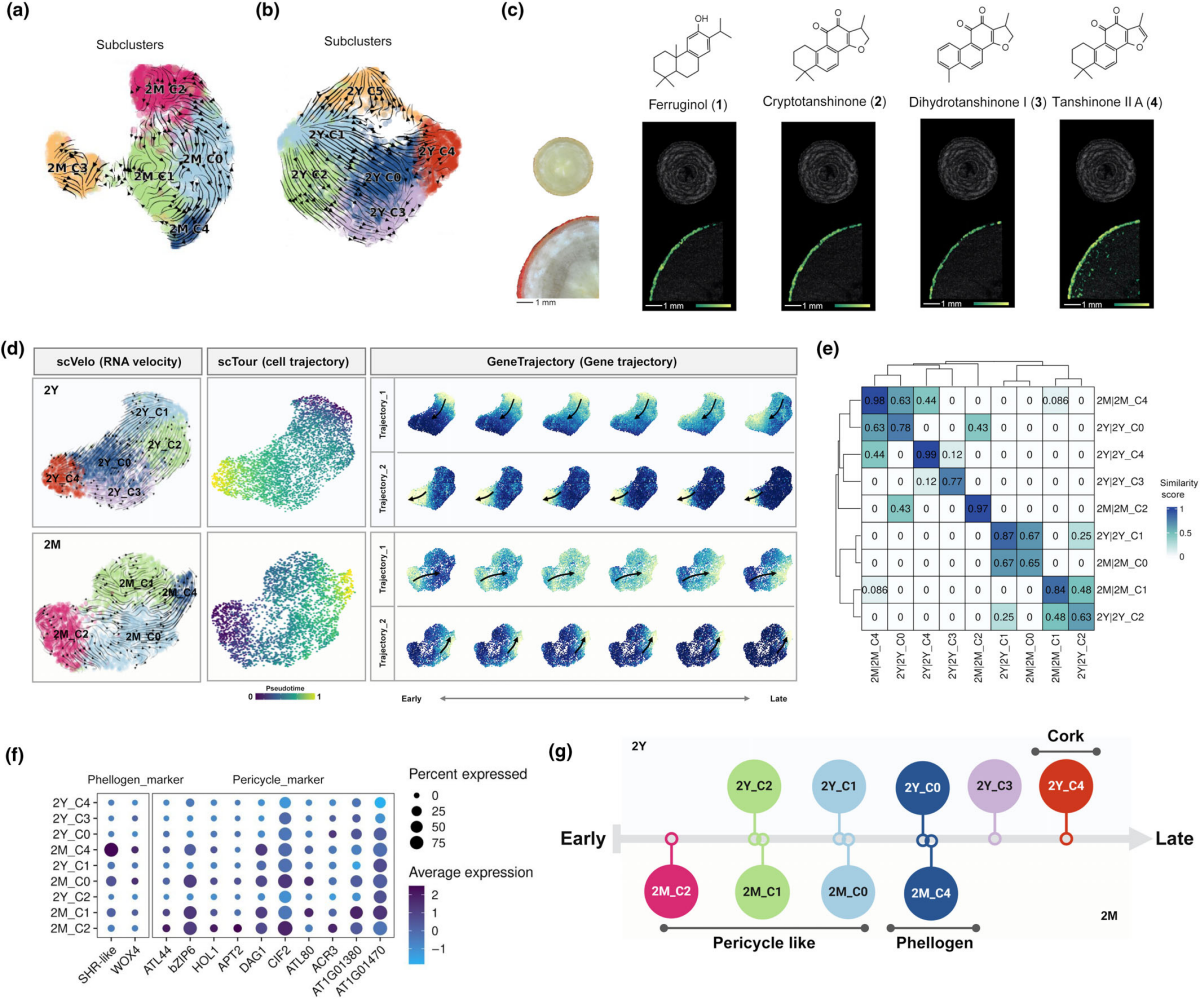
Developmental trajectories and intercellular dynamics in 2M and 2Y *Salvia miltiorrhiza* plants. (a, b) The cellular trajectories of 2M and 2Y. (c) Visualization of the spatial distribution of representative tanshinones in roots from 2‐month‐old and 2‐yr‐old plants. Numbers in the figure indicate the compounds: ferruginol (1), cryptotanshinone (2), dihydrotanshinone I (3), tanshinone IIA (4). Images are representative of three independent biological replicates showing similar distribution patterns. (d) The RNA velocity analysis of periderm‐related cells at 2M and 2Y, with epidermis excluded. (e) Correlation analysis of periderm‐related cell subsets of 2M and 2Y. (f) Markers for phellogen were derived from literature, whereas markers for pericycle were identified by comparing Arabidopsis marker genes from public databases. (g) Combined schematic diagram of cell subclusters between 2M and 2Y samples.

To investigate developmental relationships between subclusters, we reconstructed pseudotime trajectories for each sample. Two distinct branches were observed in both cases. Cellular trajectories in 2M were initiated at 2M_C2, moving through 2M_C1 and 2M_C0 before culminating at 2M_C4. In 2Y, trajectories started from 2Y_C2 and progressed sequentially through 2Y_C1, 2Y_C0, and 2Y_C3, ultimately terminating at 2Y_C4 (Fig. 4a,b).

According to AP‐MALDI‐MSI of *S. miltiorrhiza* roots at 2M and 2Y stages, tanshinone diterpenoids were barely detectable in the 2M roots, indicating limited biosynthesis and accumulation during early development. By contrast, 2Y roots exhibited marked accumulation of these compounds, predominantly localized in the outermost cork layer (Fig. 4c). A region‐guided LC‐MS was conducted with separate measurements on the cork and its inner tissues from 2Y roots, which showed higher levels of cryptotanshinone, dihydrotanshinone I, and tanshinone IIA in cork tissues compared with inner tissues (Fig. S11). These findings are consistent with the spatial distribution observed by AP‐MALDI‐MSI and further support the association of tanshinone accumulation with cork tissues in mature roots.

Given that 2Y_C4 was identified as the outermost cell layer associated with tanshinone biosynthesis, we specifically focused on the differentiation trajectories from 2M_C2 to 2M_C4 and from 2Y_C1 to 2Y_C4, together with RNA velocity and gene trajectory analyses, which elucidated the directionality of transcriptional dynamics and reinforced the inferred lineage relationships (Fig. 4d). By integrating these three approaches, we achieved a more comprehensive and robust understanding of the developmental progression and cell fate determination within the periderm lineage. To explore the intrinsic network of cell clusters across different developmental stages, we performed correlation analysis on the cell subclusters of the 2M and 2Y samples. The results revealed several pairs of subclusters with high similarity, specifically 2M_C0 and 2Y_C1, 2M_C1 and 2Y_C2, 2M_C4 and 2Y_C0 (Fig. 4e).

Using homologs of established marker genes, the periderm subclusters were further identified. Subclusters 2M_C4 and 2Y_C0 were identified as phellogen, which was supported by the expression pattern of specific marker gene, *SHR‐like*, previously reported as a phellogen‐specific marker in *Populus trichocarpa* (Miguel *et al*., 2016; C. Yu *et al*., 2023), which showed a high expression in 2M_C4, while known pericycle markers were distinctly and highly expressed in subclusters 2M_C0, 2M_C1, 2M_C2, 2Y_C1, and 2Y_C2 (Fig. 4f; Table S7). Overall, these results clearly depict the developmental process of periderm, which serves as the primary site for tanshinone biosynthesis judged from different analytical dimensions, from the early stage 2M_C2 to the mature 2Y_C4, that is from pericycle to cork, alongside the formation of phellogen (Fig. 4g).

### SmCYP76AK5 acts in tanshinone biosynthesis

**Fig. 5:**
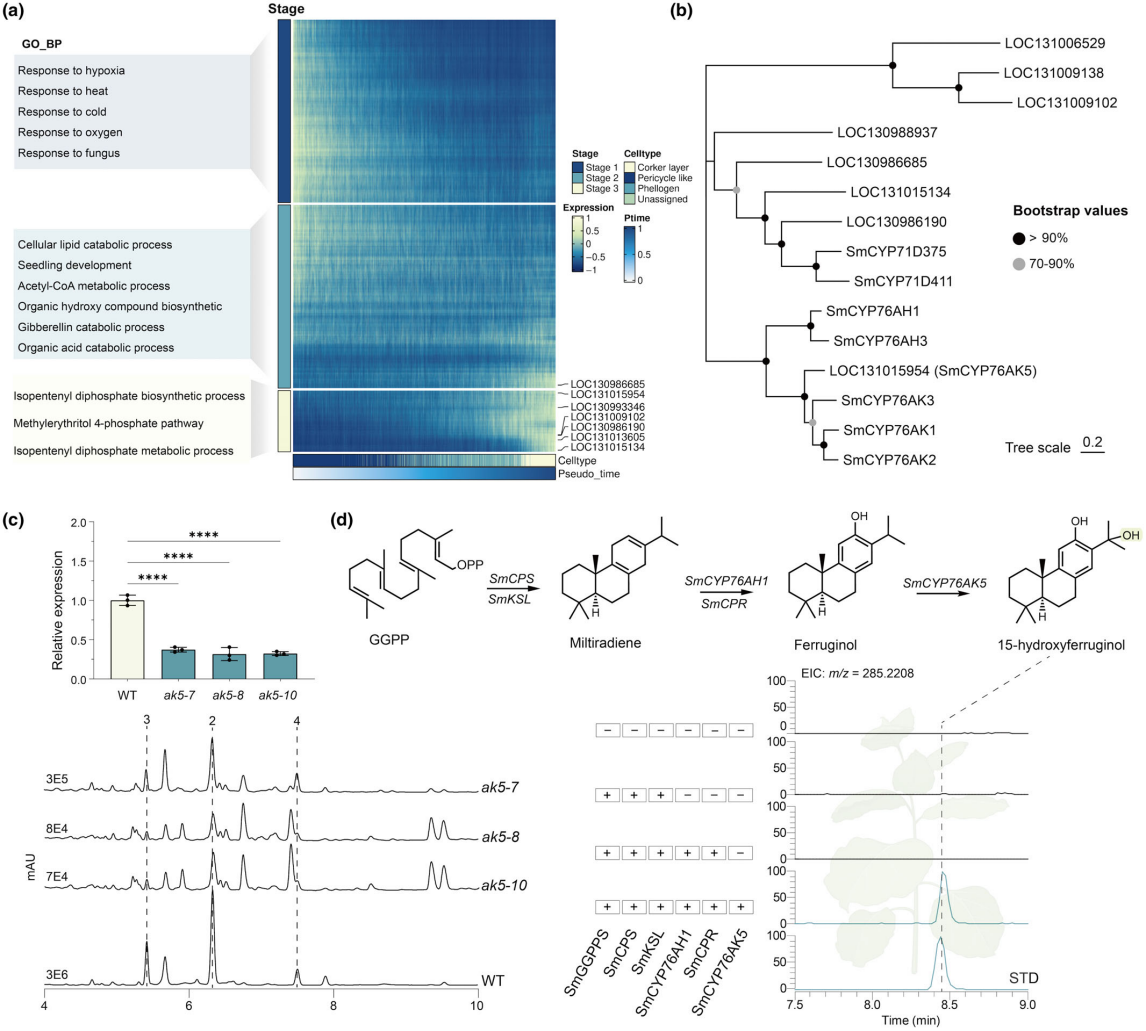
Discovery and functional characterization of SmCYP76AK5 involved in tanshinone biosynthesis. (a) Heatmap of gene expression across cell types in chronological order, with enriched pathways indicated (partial display) (Supporting Information Tables S8–S10). (b) Phylogenetic tree of cytochrome P450 family genes identified from highly expressed genes in Stage 3 based on annotations. (c) Reverse transcription quantitative polymerase chain reaction analysis and chromatograms of *SmCYP76AK5* knockout lines of *Salvia miltiorrhiza* hairy roots, *ak5‐7*, *ak5‐8*, and *ak5‐10*, generated with CRISPR‐Cas9. Data represent mean ± SD of three biological replicates (independent transgenic events), with three technical replicates per biological replicate. ****, *P* < 0.001 (one‐way ANOVA with Tukey's test). Numbers in the figure indicate the compounds: 2, cryptotanshinone; 3, dihydrotanshinone I; 4, tanshinone IIA. (d) Characterization of SmCYP76AK5. The enzymes were expressed in combination, as indicated, in *Nicotiana benthamiana* leaf. STD, standard of 15‐hydroxyferruginol.

To analyze metabolic steps, which contribute to the biosynthesis of tanshinones, we performed KEGG enrichment on the 2Y sample, based on RNA velocity time points (Fig. 5a). The enriched pathways and associated genes were systematically classified according to primary growth, morphogenesis, and organ development. To better understand the correspondence between the cell‐level pseudotime trajectory and gene expression dynamics, we constructed a combined heatmap with three continuous developmental stages: pericycle stage, intermediate transition stage, and cork layer maturation stage, which proposed a developmental overview of periderm‐related cells. Building on this trajectory analysis, we additionally applied tradeSeq to define pseudotime‐dependent gene modules and performed module‐wise gene‐set enrichment, which supported a transition from early stress/redox regulation to late enzyme programs enriched for CYPs/oxidoreductase families during cork maturation (Fig. S12). Clearly, this maturation stage only appeared in the 2Y sample. In the maturation stage, there were a few genes annotated as CYPs potentially involved in tanshinone biosynthesis that were identified and visualized on the diagrams (Fig. 5a). Furthermore, by constructing a phylogenetic tree with known CYPs as references, we retrieved a comprehensive set of candidate CYPs (Fig. 5b).

Among all these candidates in the phylogenetic tree, we knocked out the gene *SmCYP76AK5* using CRISPR‐Cas9 (Fig. S13). RT‐qPCR analysis in the *S. miltiorrhiza* hairy root system showed markedly reduced transcript levels of *SmCYP76AK5* in the knockout lines (*ak5‐7*, *ak5‐8*, and *ak5‐10*). Targeted metabolite profiling further revealed a significant reduction of tanshinones, including dihydrotanshinone I, cryptotanshinone, and tanshinone IIA, in roots of the knockout lines compared to the wild‐type (Fig. 5c). To rule out off‐target CRISPR effects, a genetic complementation assay was performed. *SmCYP76AK5* was expressed with the 35S promoter in the knockout hairy root *ak5‐8* (Fig. S14a); multiple independent complementation lines (*ak5‐8/AK5‐1*, *ak5‐8/AK5‐2*, and *ak5‐8/AK5‐5*) significantly restored accumulations of dihydrotanshinone I, cryptotanshinone, and tanshinone IIA (Fig. S14b).

To identify the activity of SmCYP76AK5 in tanshinone biosynthesis, we expressed several combinations of genes in *N. benthamiana* leaves, including the cDNAs of *SmGGPPS*, *SmCPS*, *SmKSL*, *SmCYP76AH1*, *SmCPR*, and *SmCYP76AK5*. LC‐MS results (Fig. 5d) revealed a peak with the same retention time and MS spectra as 15‐hydroxyferruginol standard; the structure was further confirmed by 1D nuclear magnetic resonance (NMR) spectra (Fig. S15). The enzymatic activity was further determined in a yeast feeding assay, in which the expression of SmCYP76AK5 in *S. cerevisiae* WAT11 supplemented with ferruginol yielded the same compound (Fig. S16). Subcellular localization analysis showed that SmCYP76AK5 colocalized with an endoplasmic reticulum (ER) marker resistance to phytophthora parasitica 1 (Pan *et al*., 2016) (Fig. S17), indicating that the hydroxylation reaction likely occurs on the ER membrane. All these results support that SmCYP76AK5 hydroxylates ferruginol to 15‐hydroxyferruginol, a possible intermediate of the tanshinone biosynthetic network.

## Discussion

### BIPACT, an analytical method for multisample integration that effectively eliminates batch effects

In this study, we generated the first scRNA‐seq dataset of the storage root of the perennial herb *S. miltiorrhiza*. We initially attempted to integrate the 2M and 2Y scRNA‐seq datasets for joint annotation; however, a significant batch effect between the samples hindered effective integration using existing methods. This prompted us to revise our strategy by annotating the 2M and 2Y samples separately and integrating them using existing annotations (Luecken & Theis, 2019), for which we designed a semisupervised sample integration algorithm based on deep learning, BIPACT. Inspired by the BERMUDA algorithm (Wang *et al*., 2019; X. Yu *et al*., 2023), BIPACT effectively integrates batches with differing cell population compositions by optimizing the Maximum Mean Discrepancy of highly reliable shared cell types. When applied to the *S. miltiorrhiza* data, BIPACT leverages credible shared cell type information to unveil intrinsic relationships between cell types while mitigating batch effects, substantially outperforming existing algorithms.

### ScRNA‐seq technology advances the understanding of perennial herb root development

**Fig. 6:**
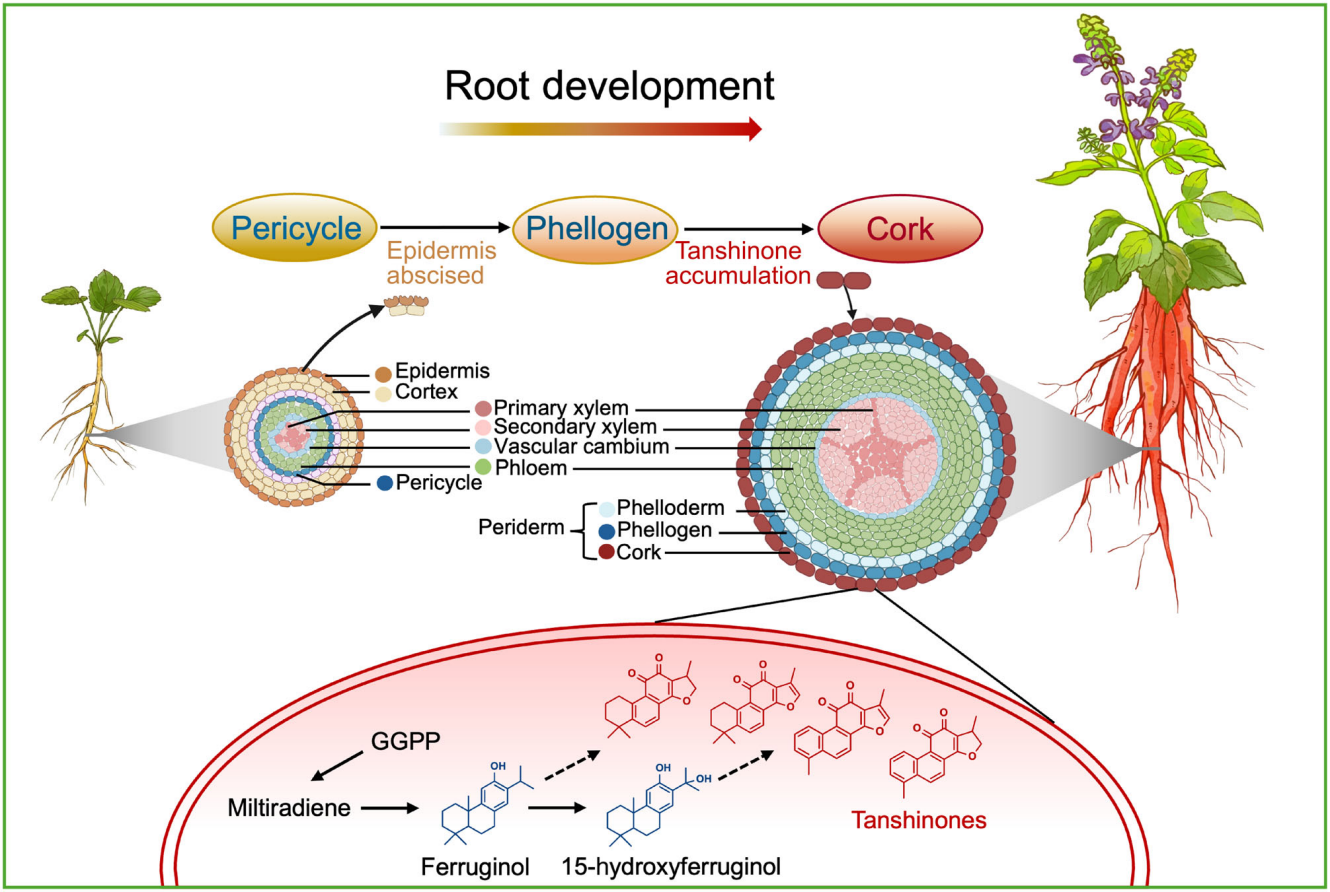
Biosynthesis and accumulation of tanshinones in the periderm of *Salvia miltiorrhiza* during secondary root growth. The periderm originates from the pericycle and differentiates into layers of phellogen (cork cambium), which gives rise to the cork (phellem). During this process, genes encoding enzymes in the tanshinone biosynthetic pathway are expressed throughout the periderm and peak in the cork layer, in which tanshinones accumulate to the maximum level. Solid arrows represent the biosynthetic pathways elucidated, while dashed arrows represent unknown pathways. Parts of this figure were created in BioRender (https://BioRender.com/27abyx0).

By combining gene trajectories and pseudotime analyses with cell‐type annotation, we constructed a periderm development atlas. Both 2M and 2Y data exhibited a dominant trajectory, excluding 2M_C3 (Fig. S18) and 2Y_C5 (Fig. S19), which were identified as epidermal cells based on marker gene expression. In most eudicots and gymnosperms, the periderm replaces the epidermis during secondary growth, protecting the vasculature from biotic and abiotic stresses (Xu *et al*., 2015; Lyu *et al*., 2025). During root secondary growth, the epidermis and cortex are abscised, while the endodermis undergoes programmed cell death, ultimately leading to cell death and tissue peeling from mature roots (Wunderling *et al*., 2018). Consistently, we observed that epidermal cells of *S. miltiorrhiza* roots were abscised during development from 2M to 2Y, while the periderm arose from inner tissues to form an external barrier, likely associated with stress responses (Fig. 6).

Based on cell‐type identification using marker genes, we found that the dominant pathways in 2M root were mainly associated with transporters and signaling molecules involved in environmental stress responses, whereas the 2Y root was enriched in metabolic pathways related to biotic resistance. The dominant trajectory corresponded to the key cell populations responsible for biosynthesis of tanshinones, with terminal cells exhibiting high expression of the tanshinone‐related genes. In roots, the cork (phellem) cells are rich in suberin, lignin, and waxes, forming the periderm barrier and providing protection against pathogens (Vishwanath *et al*., 2013; Wunderling *et al*., 2018). Tanshinones, as secondary metabolites, accumulate in the outermost layer of the root and are biosynthesized during periderm development, with their production influenced by various environmental stresses, suggesting a protective role of these furan‐bearing diterpenoids.

Using cell‐level pseudotime trajectory and gene expression dynamics, we identified three characteristic stages of periderm development: the pericycle stage, an intermediate transition stage, and the phellem stage (cork layer maturation). One of the key characteristics of the phellem is its high tanshinone content, resulting from the expression of tanshinone biosynthetic genes in the cork layer. To elucidate the tanshinone biosynthetic pathway, we selected CYPs specifically expressed in the cork layer and identified a previously uncharacterized enzymatic activity of SmCYP76AK5, which catalyzes the hydroxylation of ferruginol at C‐15. In *Tripterygium wilfordii*, CYP81AM1 was shown to specifically catalyze the formation of 15‐hydroxydehydroabietic acid but was inactive toward abietatriene or dehydroabietinol, suggesting that this specificity may be related to the presence of the carboxyl group (J. Wang *et al*., 2021). In *Salvia* species, members of the CYP76AK subfamily, including CYP76AK1 and CYP76AK6–8, have been demonstrated to catalyze the C‐20 oxidation (Guo *et al*., 2016; Scheler *et al*., 2016; Hu *et al*., 2023). The knockout of candidate genes in medicinal plants via CRISPR‐Cas9 provides an effective approach for studying the function of enzymes in metabolic pathways (Liu *et al*., 2025). In the present study, we identified CYP76AK5 in *Salvia* as a C‐15 hydroxylase. Tanshinones decreased in the CRISPR‐edited hairy roots of *SmCYP76AK5*; however, LC‐MS analysis did not reveal a substrate accumulation, suggesting a redirection of the diterpenoid flux within the metabolic grid. SmCYP76AK5 may participate in multiple reactions that finally lead to tanshinones of varied structures. Abietane‐type diterpenes, characterized by a tricyclic ring system, include ferruginol analogues, exhibit significant cytotoxicity (González, 2014; Shao *et al*., 2023). Our results expand the functional repertoire of the SmCYP76AK subfamily and highlight a previously uncharacterized branch of the diterpene biosynthetic pathway in *S. miltiorrhiza*, which also provides a foundation for future metabolic engineering efforts aimed at producing bioactive compounds in heterologous hosts.

## Competing interests

None declared.

## Author contributions

LY and JH conceived the project. LL, LG and Z‐YW designed the experiments. YL carried out the single‐cell sequencing. Z‐YW performed metabolite analysis. LG completed single‐cell data processing. XL contributed to gene editing experiments. LL and HJ performed functional characterization experiments. HL and ZS performed complementation and transformation experiments. W‐JC performed microscopy assays. Y‐BH collected and cultured plants. YK and XF assisted with LC‐MS analysis. J‐JX and H‐PC assisted in the drawing of the schematic diagram. LL, LG and Z‐YW analyzed the data and wrote the first draft of the manuscript. LL, LG, Z‐YW and YL contributed equally to this article. JG, Y‐HH, and KC assisted in revising the manuscript. All authors contributed to manuscript revision. X‐YC supervised the project. All authors read and approved the final manuscript.

## Disclaimer

The New Phytologist Foundation remains neutral with regard to jurisdictional claims in maps and in any institutional affiliations.

## Supporting information


**Fig. S1** TICs of *Salvia miltiorrhiza* roots across developmental stages.
**Fig. S2** Trypan blue staining of protoplasts.
**Fig. S3** Conventional cell type annotation of 2M and 2Y samples.
**Fig. S4** Overview of the DsAno pipeline and evaluation of its annotation performance.
**Fig. S5** MetaNeighbor analysis of cross‐sample similarity.
**Fig. S6** Overview of the BIPACT computational pipeline.
**Fig. S7** Benchmarking of BIPACT against seven integration methods.
**Fig. S8** Comparative evaluation of BIPACT integration performance in *Salvia miltiorrhiza* scRNA‐seq data.
**Fig. S9** The biosynthetic pathway of tanshinones in *Salvia miltiorrhiza*.
**Fig. S10** Expression patterns of genes in the MVA and MEP tanshinone biosynthetic pathways across 2M and 2Y root clusters.
**Fig. S11** LC‐MS analysis of tanshinone accumulation in cork and inner root tissues of 2Y *Salvia miltiorrhiza*.
**Fig. S12** Gene‐set enrichment of trajectory‐dependent gene modules.
**Fig. S13** CRISPR‐Cas9 mediated knockout of *CYP76AK5*.
**Fig. S14** Genetic complementation of *SmCYP76AK5* knockout lines and tanshinone accumulation rescue.
**Fig. S15** 1D NMR spectra of 15‐hydroxyferruginol standard.
**Fig. S16** Characterization of CYP76AK5 in yeast.
**Fig. S17** Subcellular localization of SmCYP76AK5‐GFP in transiently expressed *Nicotiana benthamiana* leaves by confocal microscopy.
**Fig. S18** Pathway enrichment of the 2M_C3 gene module.
**Fig. S19** Pathway enrichment of the 2Y_C5 gene module.
**Methods S1** BIPACT algorithm.

**Table S1** Primers used in this study.
**Table S2** Single‐cell RNA sequencing and quality‐control statistics.
**Table S3** Gene nomenclature and synteny‐supported annotations.
**Table S4** Marker genes used for cell type annotation.
**Table S5** Candidate and characterized genes involved in tanshinone biosynthesis.
**Table S6** Differentially expressed genes in periderm‐related subclusters.
**Table S7** Marker genes for identifying pericycle‐ and phellogen‐related cells.
**Table S8** Pseudotime‐associated gene lists across periderm developmental stages.
**Table S9** Stage‐specific gene lists used for developmental stage analysis.
**Table S10** Enriched pathways associated with mature (2Y) root tissues.
Please note: Wiley is not responsible for the content or functionality of any Supporting Information supplied by the authors. Any queries (other than missing material) should be directed to the *New Phytologist* Central Office.

## Data Availability

The BIPACT scripts are available in the release package on GitHub (https://github.com/Hao‐Zou‐lab/BIPACT). Single‐cell RNA‐seq raw data of this study have been deposited in the Genome Sequence Archive of the China National Genomics Data Center (https://ngdc.cncb.ac.cn/) under accession no. CRA029136 and are publicly available.
