# Supplementary material for: Cross‐stage single‐cell and spatial metabolome analyses reveal periderm specialization and tanshinone biosynthesis in Salvia miltiorrhiza roots
Source: New Phytol. 2026 May 21;251(3):1267–82. doi: 10.1111/nph.71285 (PMC13326519; doi:10.1111/nph.71285)
Supplement: Supplementary file 1 — Fig. S1 TICs of Salvia miltiorrhiza roots across developmental stages. Fig. S2 Trypan blue staining of protoplasts. Fig. S3 Conventional cell type annotation of 2M and 2Y samples. Fig. S4 Overview of the DsAno pipeline and evaluation of its annotation performance. Fig. S5 MetaNeighbor analysis of cross‐sample similarity. Fig. S6 Overview of the BIPACT computational pipeline. Fig. S7 Benchmarking of BIPACT against seven integration methods. Fig. S8 Comparative evaluation of BIPACT integration performance in Salvia miltiorrhiza scRNA‐seq data. Fig. S9 The biosynthetic pathway of tanshinones in Salvia miltiorrhiza. Fig. S10 Expression patterns of genes in the MVA and MEP tanshinone biosynthetic pathways across 2M and 2Y root clusters. Fig. S11 LC‐MS analysis of tanshinone accumulation in cork and inner root tissues of 2Y Salvia miltiorrhiza. Fig. S12 Gene‐set enrichment of trajectory‐dependent gene modules. Fig. S13 CRISPR‐Cas9 mediated knockout of CYP76AK5. Fig. S14 Genetic complementation of SmCYP76AK5 knockout lines and tanshinone accumulation rescue. Fig. S15 1D NMR spectra of 15‐hydroxyferruginol standard. Fig. S16 Characterization of CYP76AK5 in yeast. Fig. S17 Subcellular localization of SmCYP76AK5‐GFP in transiently expressed Nicotiana benthamiana leaves by confocal microscopy. Fig. S18 Pathway enrichment of the 2M_C3 gene module. Fig. S19 Pathway enrichment of the 2Y_C5 gene module. Methods S1 BIPACT algorithm. [file NPH-251-1267-s002.pdf]

***New Phytologist* Supporting Information**

Article title: Cross-stage single-cell and spatial metabolome analyses reveal periderm specialization and tanshinone biosynthesis in *Salvia miltiorrhiza* roots

Authors: Licheng Liu[1, 2, 8], Lianchong Gao[3, 8], Zhi-Ying Wang[1, 4, 8], Yan Liu[5, 8], Huifeng Ju[1, 2], Huana Lin[1], Zheng Shi[1], Wen-Juan Cai[6], Xue Li[1], Yan-Bo Huang[1], Yu Kong[1], Jing-Jing Xu[1], Hong-Peng Chen[4], Xin Fang[5], Juan Guo[7], Yong-Hong Hu[1], Ke Chen[1], Xiao-Ya Chen[1, 6], Jie Hao[1]*, Lei Yang[1]*

Article acceptance date: 28 April 2026

Methods S1 BIPACT algorithm

Cell types were identified individually in each batch using the DsAno analysis pipeline, ensuring that data from each batch were processed independently and cell types were annotated separately. To determine the similarity between cell types across different batches, we employed MetaNeighbor (Crow et al., 2018) which utilizes Spearman correlation to assess the relationships based on gene expression patterns. MetaNeighbor calculates a similarity score for each pair of cell types by averaging the area under the receiver operator characteristic curve (AUROC) in cross-validation. This method provides a robust measure of similarity that is crucial for identifying and comparing cell types in multi-batch studies.

For j batches, each containing i pre-annotated cell types, where $i = 1, 2, \ldots, \mathrm{n}$, and each number represents a cell type in each batch, while $j = 1, 2, \ldots, \mathrm{n}$, represents the label of the batch, MetaNeighbor calculates the similarity scores between all cell types across batches. We denote $S_{i_1 j_1, i_2 j_2}$ as the similarity score between cell type i_1 in batch j_1 and cell type i_2 in batch j_2. A high similarity score between cell types indicates a potential match, but only those pairings that are consistently annotated by DsAno as the same type, such as xylem, and have the highest similarity scores are designated as Highly Reliable Cell Types (HRCT). This stringent criterion ensures that HRCTs are identified based on consistent, cross-batch similarity. If, for instance, both batch j_1 and batch j_2 possess a cell type named xylem, but the similarity score between xylem in batch j_1 and phloem in batch j_2 is higher than between xylem across the two batches, then xylem is not defined as HRCT. This differentiation helps in pinpointing HRCT across batches which are then labeled as 'HRCT' and distinguished from other cell types to serve as a reliable reference for cross-batch analyses in BIPACT.

$$S_{i_1 j_1, i_2 j_2} = \begin{cases} 1 & \textit{if } i_1 j_1 \textit{ and } i_2 j_2 \textit{ are HRCT} \\ 0 & \textit{otherwise} \end{cases} \qquad (1)$$

The mini-batch gradient descent algorithm commonly adopted in deep learning was used to train BIPACT. Mini-batch gradient descent is a variation of the gradient descent algorithm and is widely adopted in the field of deep learning (S. Khirirat et al., 2017). For each iteration in each epoch during the training process of BIPACT, a "mini-batch" $X = \{x1, x2, \ldots, x_m\}$ was sampled from the dataset, which contained n_{batch_size} cells from each celltype $(m = n_{batch\_size} \sum_{i=1}^{n} c_i)$. We used $n_{batch\_size} = 50$ in our experiments. Where X represents the mini-batch containing m individual samples. n is the number of cells sampled from each celltype, c is the total number of celltypes, c_i denotes the number of cells available in the i-th celltype. The loss function for training the autoencoder consisted of three parts.

Part 1. BIPACT employs a denoising variational autoencoder (DVAE) to project the original uncorrected gene expression profiles into a low-dimensional space, thereby reducing experimental artifacts across different batches.

We applied a DVAE to learn a low-dimensional representation from the X, here X refers to both the mini-batch of individual cell profiles sampled for training and, more broadly, to the scRNAseq expression matrix utilized by the DVAE. In this matrix, each row corresponds to a cell's gene expression profile, and each column represents a gene. The basic architecture of a DVAE is composed of three parts:

(i) a noise operation (B), which generates a noisy scRNAseq expression matrix X' by inducing random noises to X based on a binomial distribution:

$$X' = \mathrm{B}(X, p) \tag{2}$$

where p is the probability of zero value assignment in each row. Probability p quantifies the likelihood that any gene expression value is artificially set to zero, mimicking the dropout phenomena frequently observed in scRNAseq data.

(ii) an encoder E, which subtracts X' to a lower-dimensional subspace with a ReLU activation function:

$$\mu, \sigma = \mathrm{Encoder}(X') \tag{3}$$

$$z = \mu + \sigma \odot \epsilon \text{ where } \epsilon \sim \mathcal{N}(0,1) \tag{4}$$

where μ is the mean vector in the latent space that determines the central position of the encoded data, σ is the log-variance vector in the latent space representing the spread of the features, ϵ is a random noise vector sampled from a standard normal distribution, used to incorporate randomness as part of the reparameterization trick in variational autoencoders, and $\odot$ denotes the element-wise product (Hadamard product). z is the latent vector sampled from the encoded distribution, used subsequently in the decoding process. The encoder consists of a fully connected layer with 200 units followed by ReLU activation, projecting to mean μ and log-variance σ layers each with 20 units.

(iii) a decoder D, which reconstructs an approximation matrix X'' from the low-dimensional representations.

$$X'' = \mathrm{Decoder}(z) \tag{5}$$

where X'' is the reconstructed gene expression matrix approximating the original X, z is the latent vector containing encoded information from X'. The decoder uses a fully connected layer with 200 units followed by ReLU activation, and another layer that

maps back to the original input size with a sigmoid activation to ensure the outputs are within [0, 1].

The DVAE is optimized by balancing the reconstruction loss function, specifically the Mean Square Error (MSE), and the Kullback-Leibler (KL) divergence between the approximate posterior of the latent variables and their prior distribution. The MSE measures the difference between the original input matrix X and the reconstructed matrix X'', aiming to make the reconstructed matrix similar to the original. The KL divergence acts as a regularizer by ensuring the distribution of the latent variables $z$closely approximates the prior distribution, typically assumed to be a standard normal distribution $\mathcal{N}(0,1)$. The model can be trained using the following loss function:

$$\mathbf{Loss1}(X) = \mathrm{MSE}(X, X'') + \beta \cdot \mathrm{KL}\ \ \mathrm{Divergence}\ (\mu, \sigma) \tag{6}$$

Part 2. We have introduced two additional loss functions to jointly adjust the model parameters of the DVAE during training. First, the maximum mean discrepancy (MMD) based loss (Gretton et al., 2012) that estimates the differences in distributions among similar cell clusters in different batches. MMD is a non-parametric distance estimate between distributions based on the reproducing kernel Hilbert space (RKHS) and has proven to be highly effective in many deep transfer learning tasks(Chen et al., 2022). Since MMD does not require density estimates as an intermediate step and does not assume any parametric density on the data, it can be applied to different domains. MMD is also memory-efficient, fast to compute, and performs well on high dimensional data with low sample size. Considering the case where only a subset of the cell population is shared among batches, instead of applying MMD loss on batches entirely, we only considered the loss between pairs of similar cell types among different batches. So, the MMD-based loss can be defined as:

$$\mathbf{Loss2}(X) = \sum_{i_1 j_1, i_2 j_2} S_{i_1 j_1, i_2 j_2} \cdot \mathrm{MMD}\left(z_{i_1 j_1}, z_{i_2 j_2}\right) \tag{7}$$

Where $Z_{i,j}$ is the encoding of the input $X_{i,j}$, and $X_{i,j}$ represents the expression profiles of cells from cell type i of batch j within the mini-batch X. The MMD equals zero when the underlying distributions of the observed samples are the same. By minimizing the MMD loss between the distributions of the highly reliable cell type across batches, the DVAE can be trained to remove batch effects in the bottleneck layer.

Part 3. In order to optimize the performance of the Deep Variational Autoencoder (DVAE) and effectively manage the heterogeneity of cell type data across high-dimensional spaces, we have introduced a cosine similarity loss function, referred to as Loss3. This loss utilizes label information of cell types, adjusting the orientation of

embedding vectors to enhance proximity among vectors of the same cell type while ensuring that vectors representing different cell types are easily distinguishable:

$$\mathbf{Loss3}(X) = \sum_{i_1,j_1,i_2,j_2} w_{i_1 j_1} w_{i_2 j_2} \left(2S_{i_1 j_1, i_2 j_2} - 1\right) \cdot \text{cosine\_similarity}\left(z_{i_1 j_1}, z_{i_2 j_2}\right) \quad (8)$$

Where $w_{i_1 j_1}$ and $w_{i_2 j_2}$ are weights assigned to these cell types to balance their representation in the dataset, addressing potential imbalances in cell type prevalence across batches. $S_{i_1 j_1, i_2 j_2}$ is the similarity score, set to 1 if cell types i_1 from batch j_1 and i_2 from batch j_2 are annotated consistently as the same type across these batches and meet the criteria for being considered Highly Reliable Cell Types (HRCT), otherwise, it is set to 0. This score ensures that the model's training focuses on enhancing the similarity of vectors representing the same cell type across batches while differentiating between those that do not meet the HRCT criteria.

In summary, the total loss function on a mini-batch can be written as:

$$\mathbf{Loss_{Total}}(X) = \mathbf{Loss1}(X) + \alpha * \mathbf{Loss2}(X) + \beta * \mathbf{Loss3}(X) \quad (9)$$

By minimizing the total loss, the trained DVAE can effectively reduce noise, align the distribution of cell types across different batches, and enhance the distinctiveness of cell type representations in the learned embedding space.

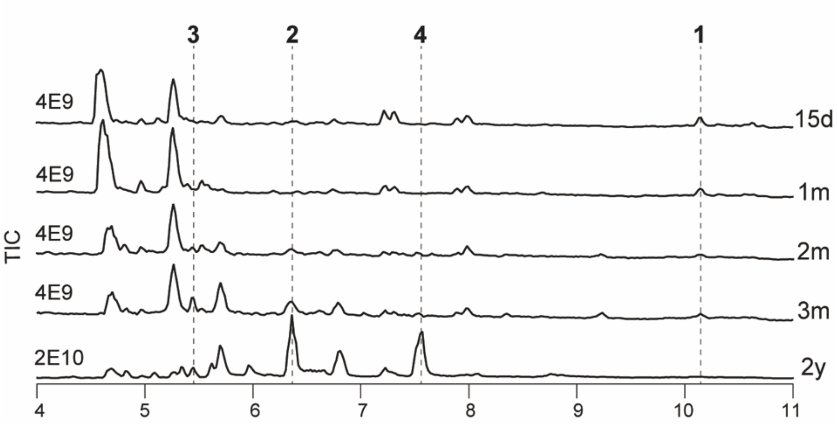

Fig. S1. Total ion chromatograms (TICs) of *S. miltiorrhiza* roots across developmental stages. TICs were generated from LC-MS analysis of root samples at 15 days (15 d), 1 month (1 m), 2 months (2 m), 3 months (3 m), and 2 years (2 y) of age. Numbers indicate representative tanshinone-related compounds: 1, ferruginol; 2, cryptotanshinone; 3, dihydrotanshinone I; 4, tanshinone IIA.

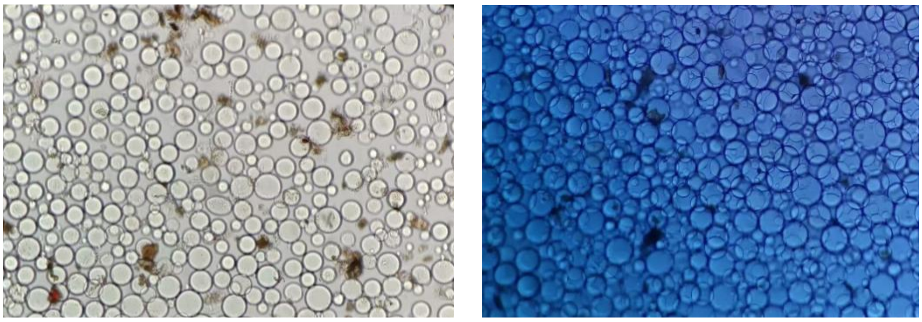

Fig.S2 Protoplast staining before (left) and after (right) by trypan blue.

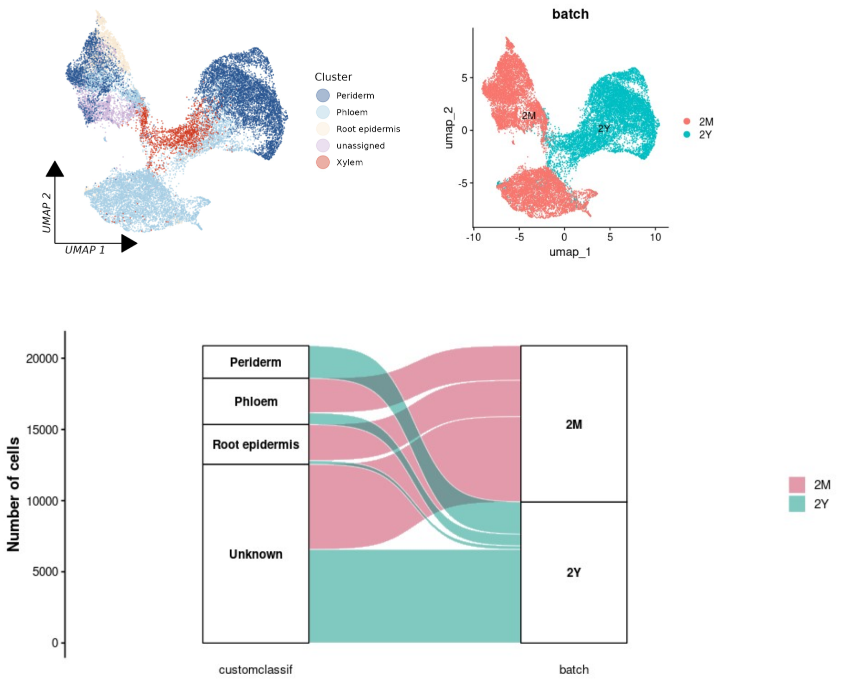

Fig. S3 Traditional annotation results of two samples. UMAP visualization of scRNA-seq data after Harmony integration, colored by annotated cell types and sample origin (2-month-old, 2M; 2-year-old, 2Y). Despite integration, cells from the two samples remain partially separated. Alluvial plot showing the correspondence between annotated cell types and sample origin. A large proportion of cells from the 2Y sample could not be assigned to known cell types using a standard marker-based annotation approach and were classified as "Unknown", highlighting the limitations of this strategy in resolving cell identities across samples.

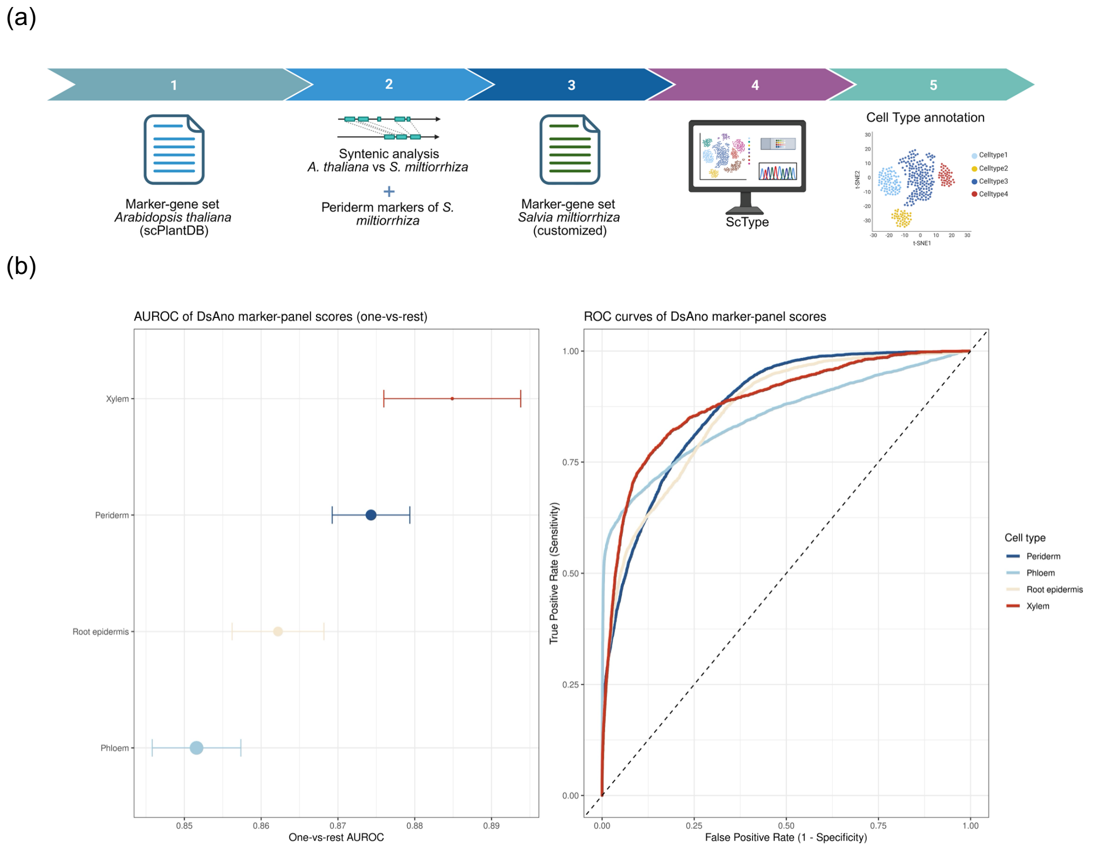

Fig. S4 Flowchart of the DsAno pipeline and AUROC scores. (a) The workflow diagram of the DsAno pipeline. Marker-gene set construction (Steps 1-3), where a customized *Salvia miltiorrhiza* reference set is generated by mapping Arabidopsis markers (from scPlantDB) via syntenic analysis and incorporating specific periderm markers; and automated cell type annotation (Steps 4-5), utilizing the ScType algorithm to assign cell identities based on the customized marker set. (b) The figure presents the one-vs-rest AUROC scores (left) and the corresponding ROC curves (right) for distinct cell type subclusters, demonstrating the predictive performance and robustness of the assigned marker panels.

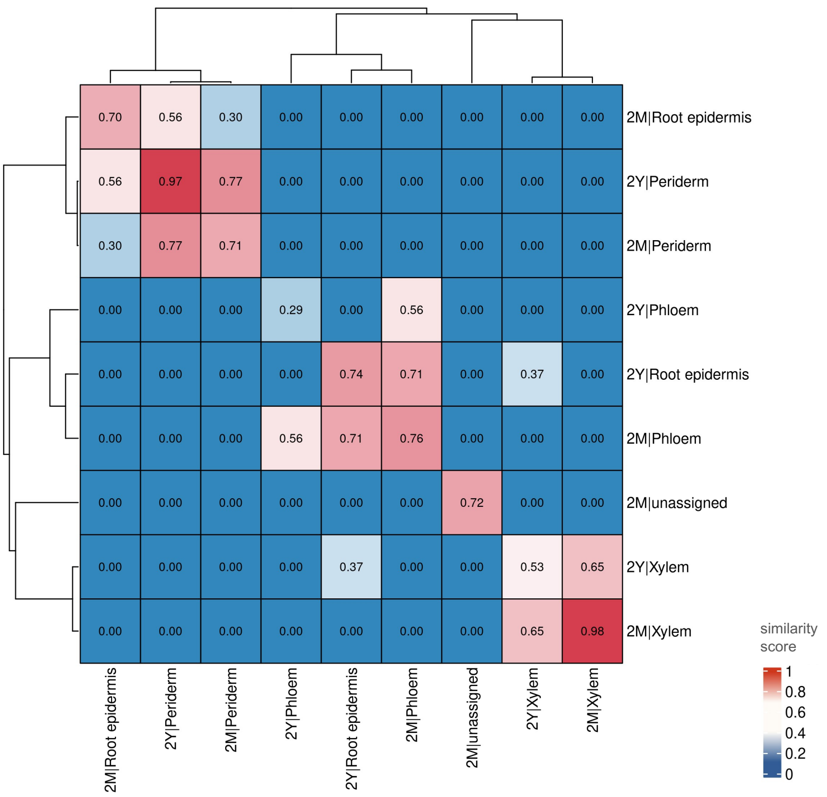

Fig. S5 Evaluation of cell type conservation using MetaNeighbor. The heatmap quantifies the similarity of cell type expression profiles between the 2M and 2Y datasets. The color scale represents the similarity score (AUROC), with high values indicating reproducible cell type identification across samples.

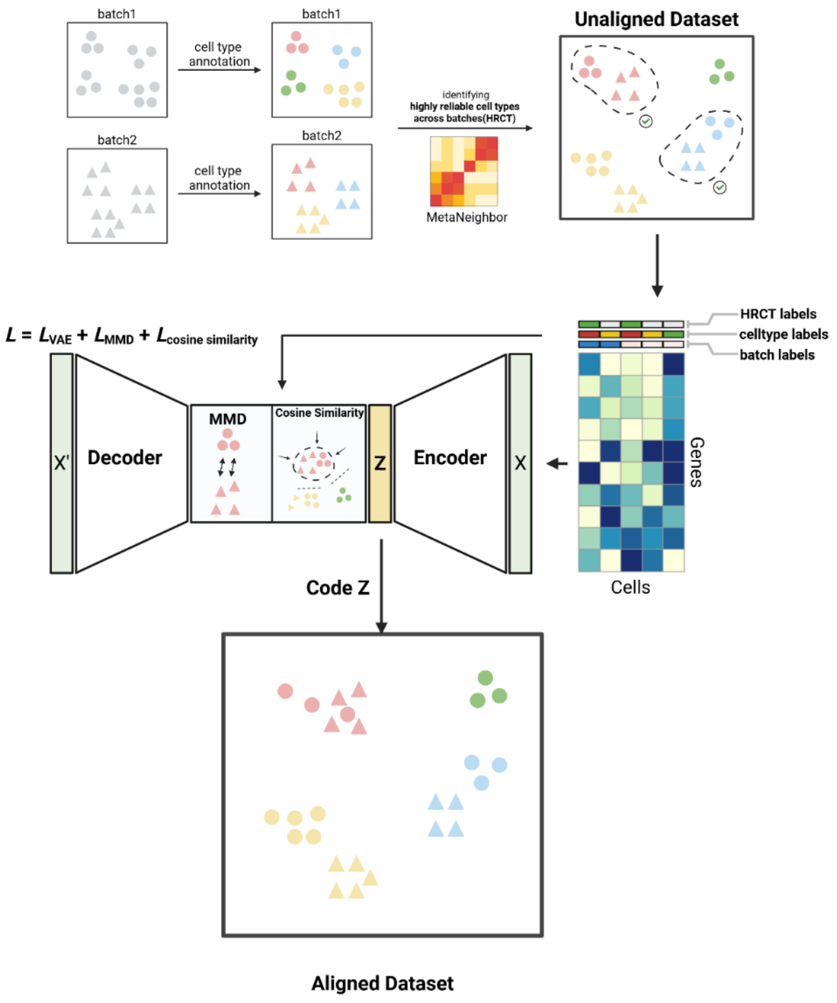

Fig. S6 Flowchart of BIPACT processing.

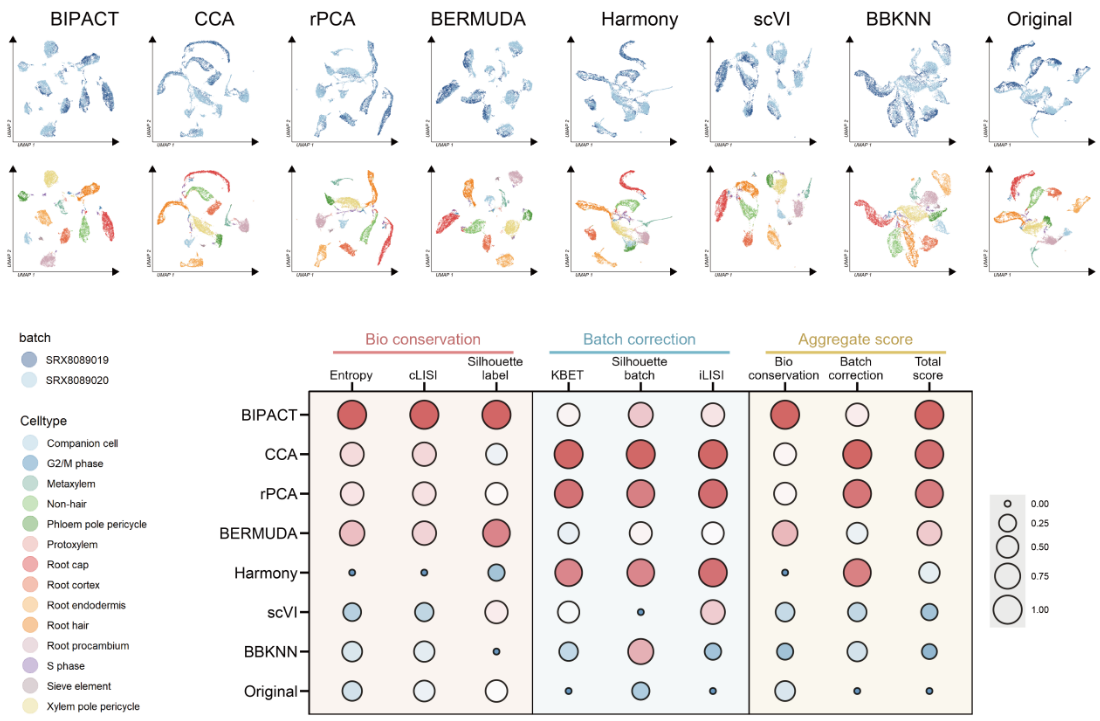

Fig. S7 Performance evaluation using a gold-standard model organism dataset with well-defined cell type annotations. Qualitative UMAP visualizations comparing BIPACT with uncorrected data (Original) and seven integration methods (CCA, rPCA, BERMUDA, Harmony, scVI, BBKNN). Quantitative assessment using metrics for biological conservation (Entropy, cLISI, Silhouette label) and batch correction (kBET, Silhouette batch, iLISI). Bubble size and color intensity indicate the normalized score (0–1). BIPACT demonstrates superior aggregate performance, validating its accuracy in preserving established biological structures while removing batch effects.

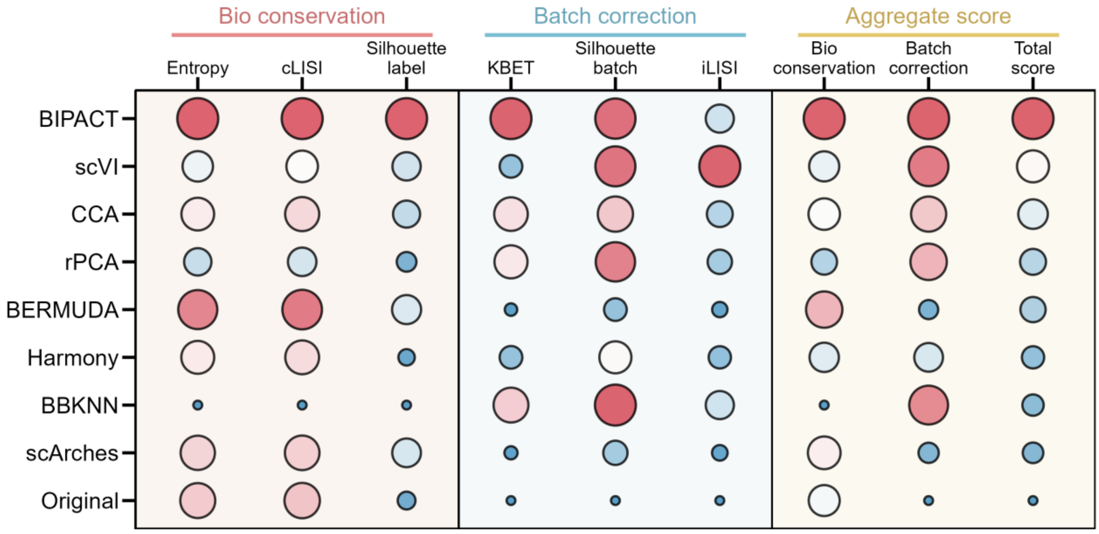

Fig. S8 Evaluation of BIPACT integration performance in a real-world dataset of *Salvia miltiorrhiza*. Comparative analysis of BIPACT versus seven established integration methods and the uncorrected data (Original) using UMAP visualization. Metrics cover biological conservation (e.g., cLISI, Silhouette label) and batch mixing (e.g., kBET, iLISI). Normalized scores (0-1) indicated by bubble aesthetics show that BIPACT achieves the highest aggregate score, effectively balancing batch correction with the preservation of complex biological structures in non-model organism data.

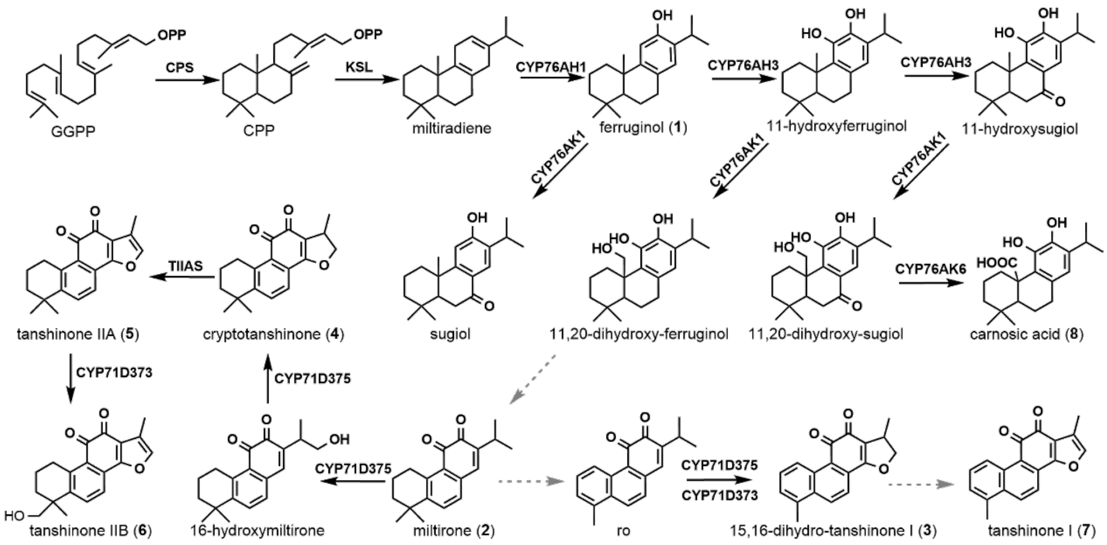

Fig. S9 The biosynthetic pathway of tanshinones in *S. miltiorrhiza*.

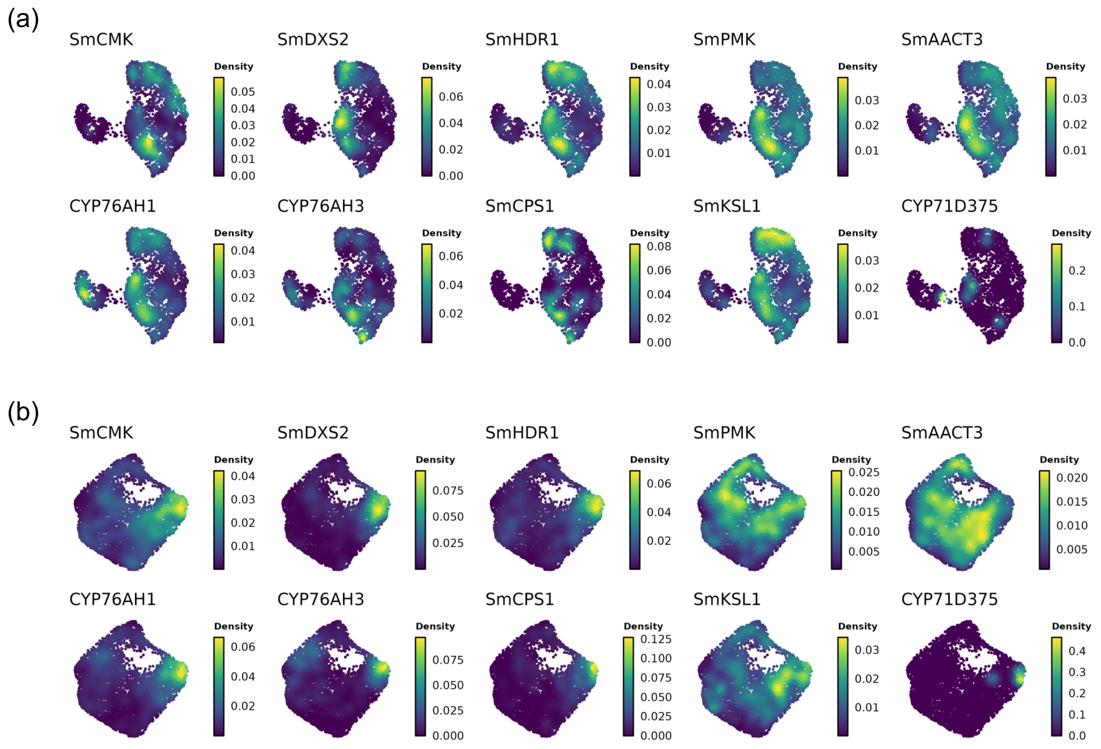

Fig. S10 Visualization of gene expression in the MVA and MEP pathways of tanshinone biosynthesis in 2M (a) and 2Y (b) clusters. Gene expression analysis of the MVA and MEP pathways revealed no distinct clustering pattern in 2M, whereas 2Y exhibited a clear spatial concentration-most genes were highly expressed in the upper-right region of the plot, corresponding to the 2Y_C4 subcluster.

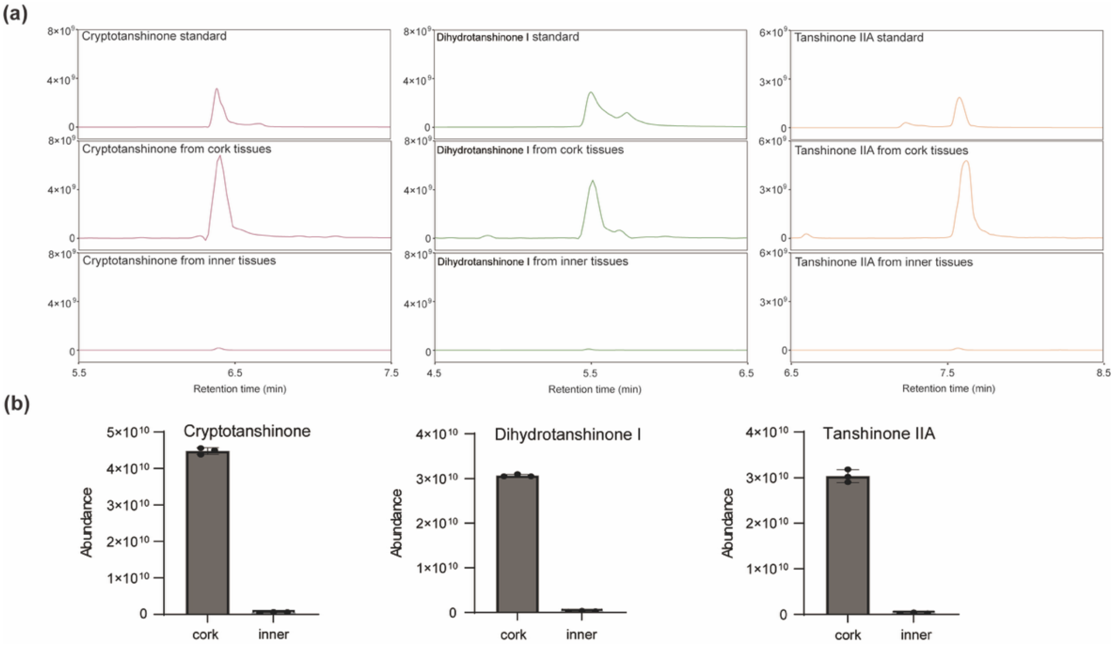

Fig. S11 Region-guided LC-MS analysis of tanshinone accumulation in cork and inner tissues of 2-year-old *S. miltiorrhiza* plants. (a) Extracted ion chromatograms (EICs) of three downstream tanshinone compounds - cryptotanshinone, dihydrotanshinone I, and tanshinone IIA - from cork and inner tissues. Colored traces correspond to each compound: cryptotanshinone (pink), dihydrotanshinone I (green), tanshinone IIA (yellow). (b) Corresponding peak areas of the three tanshinones in cork and inner tissues are shown as bar graphs. Data represent mean ± SD of three biological replicates (n = 3).

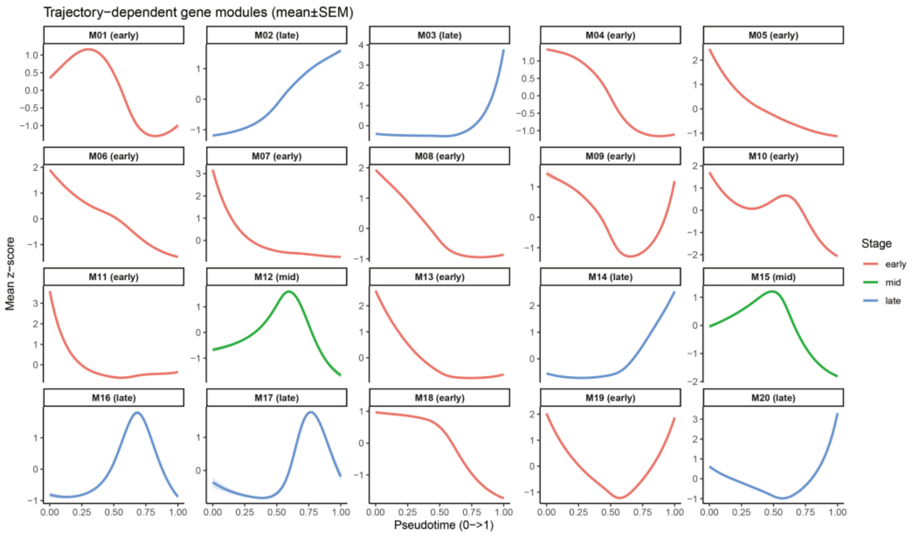

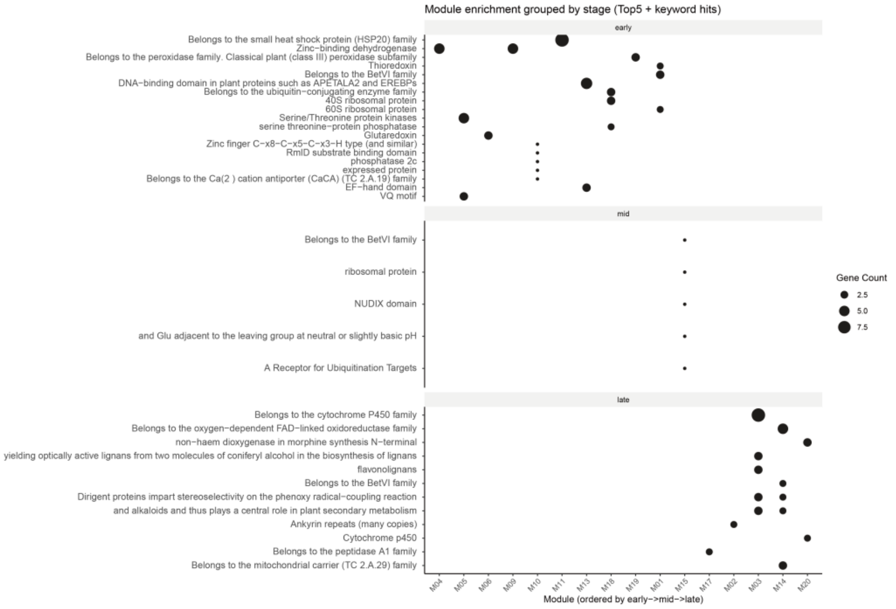

Fig. S12 Gene-set enrichment of trajectory-dependent gene modules.

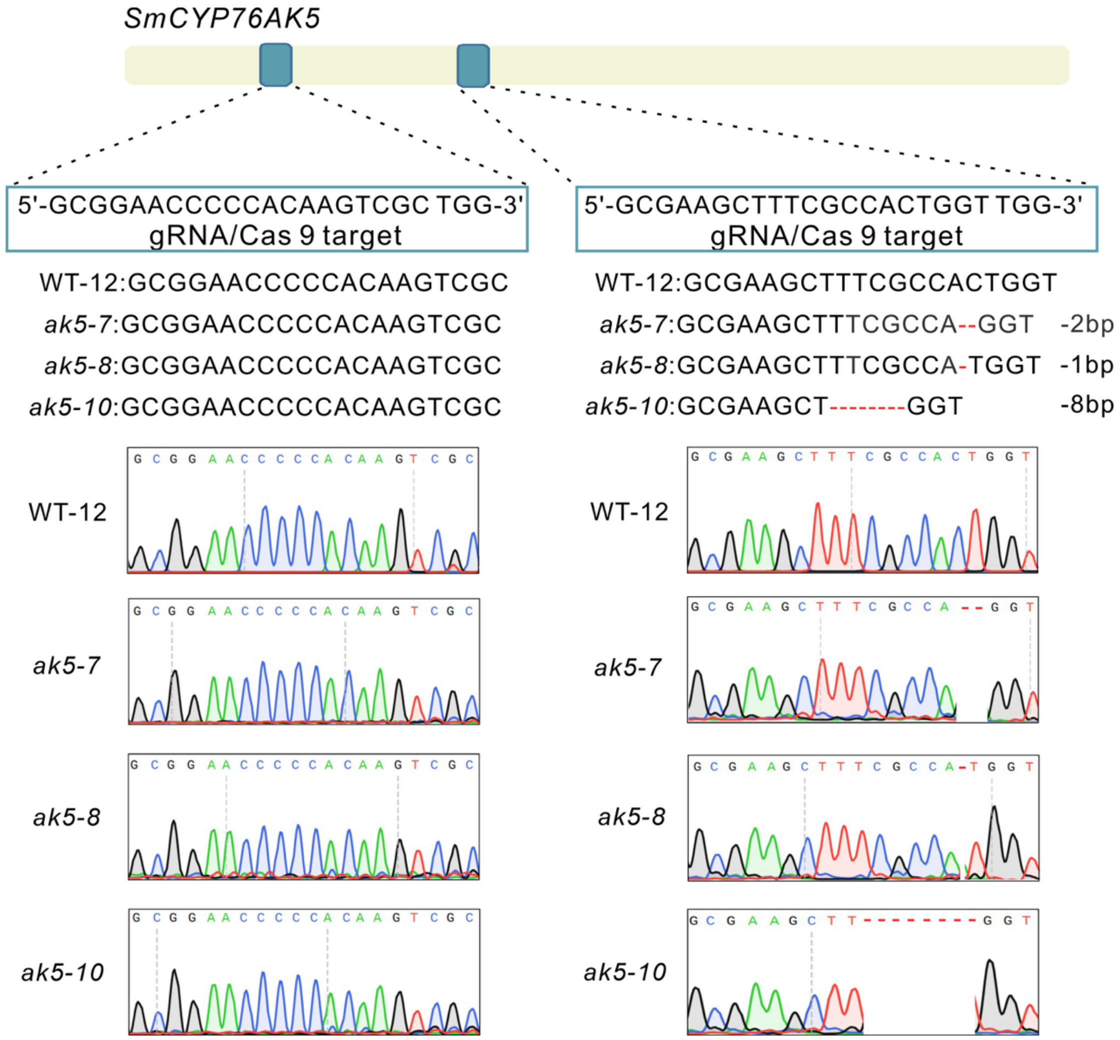

Fig. S13 CRISPR-Cas9 mediated knockout of *SmCYP76AK5*. Schematic of Golden Gate assembly strategy for dual-sgRNA construct targeting Sm*CYP76AK5* (sgRNA1: 5′-GCGGAACCCCCACAAGTCGT-TGG-3′; sgRNA2: 5′-GCGAAGCTTCCGCCACTGGTTGG-TGG-3′). Deletion efficiency at target loci confirmed by Sanger sequencing. Relative mRNA expression in knockout lines (*ak5-7*, *ak5-8* and *ak5-10*) versus wild-type (WT-12).

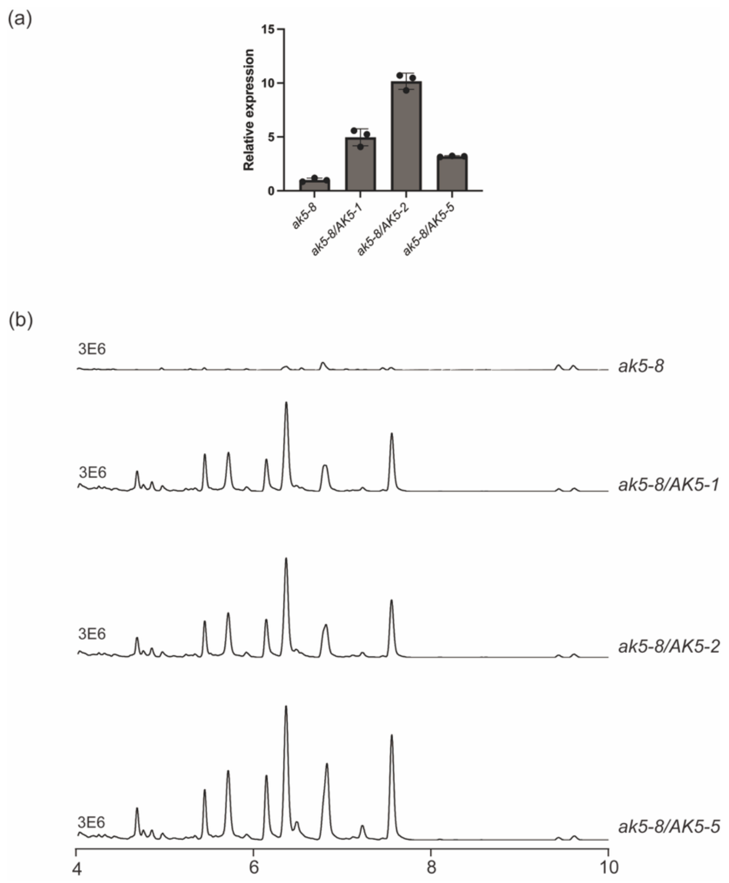

Fig.S14 Genetic complementation restores tanshinone accumulation in *SmCYP76AK5* knockout lines. (a) Relative expression levels of SmCYP76AK5 in three independent complementation lines (*ak5-8/AK5-1*, *ak5-8/AK5-2*, *ak5-8/AK5-5*) as determined by RT-qPCR. Expression levels were normalized to the internal reference gene. Data represent mean ± SD (n = 3 biological replicates). (b) Total ion chromatograms (TICs) of methanol extracts from *SmCYP76AK5* knockout (*ak5-8*), and complementation lines. Restoration of tanshinone-associated peaks in complementation lines indicates recovery of metabolic phenotype.

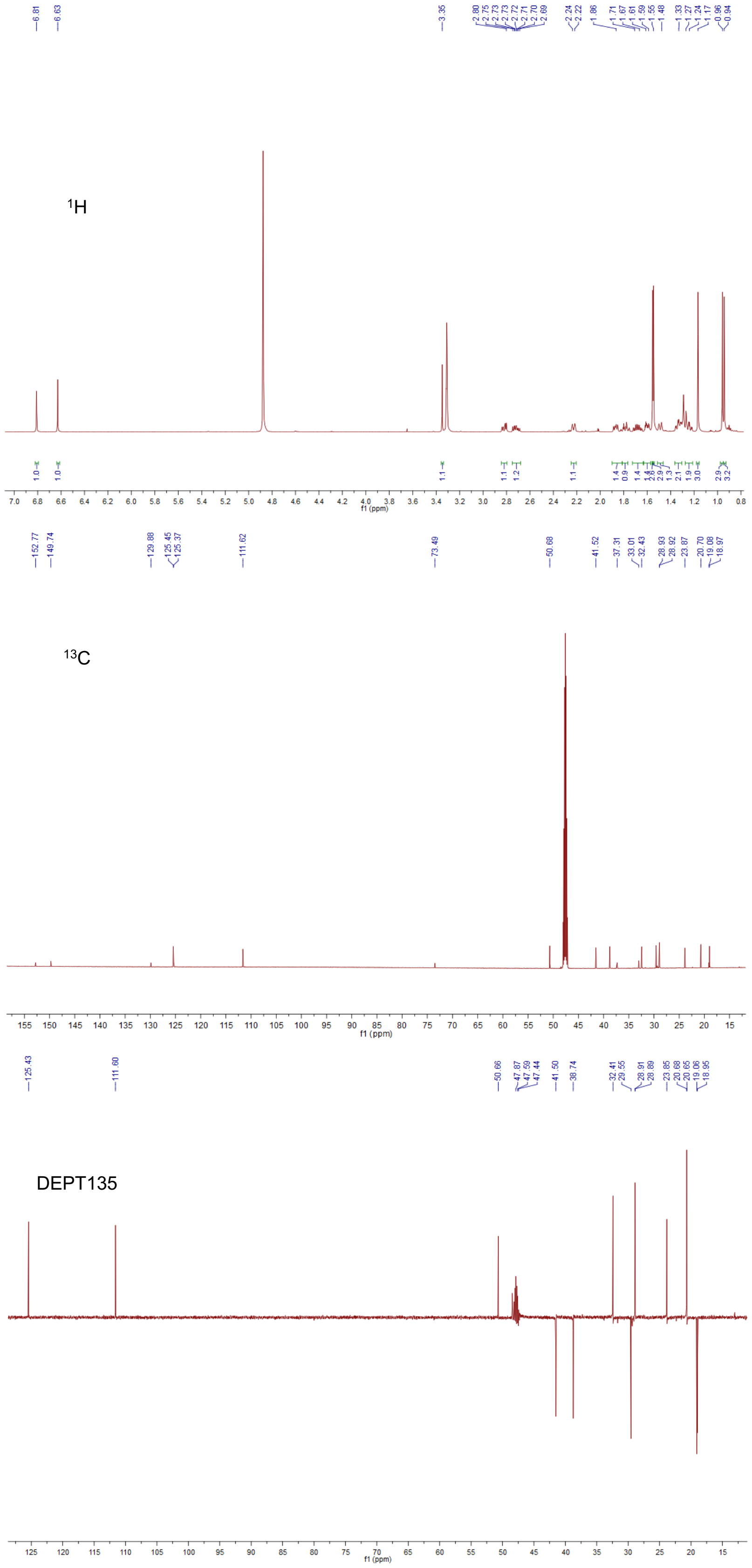

Fig. S15 1D (^{1}H, 600MHz; ^{13}C, 400MHz, in CDOH) NMR spectra of 15-hydroxyferruginol standard.

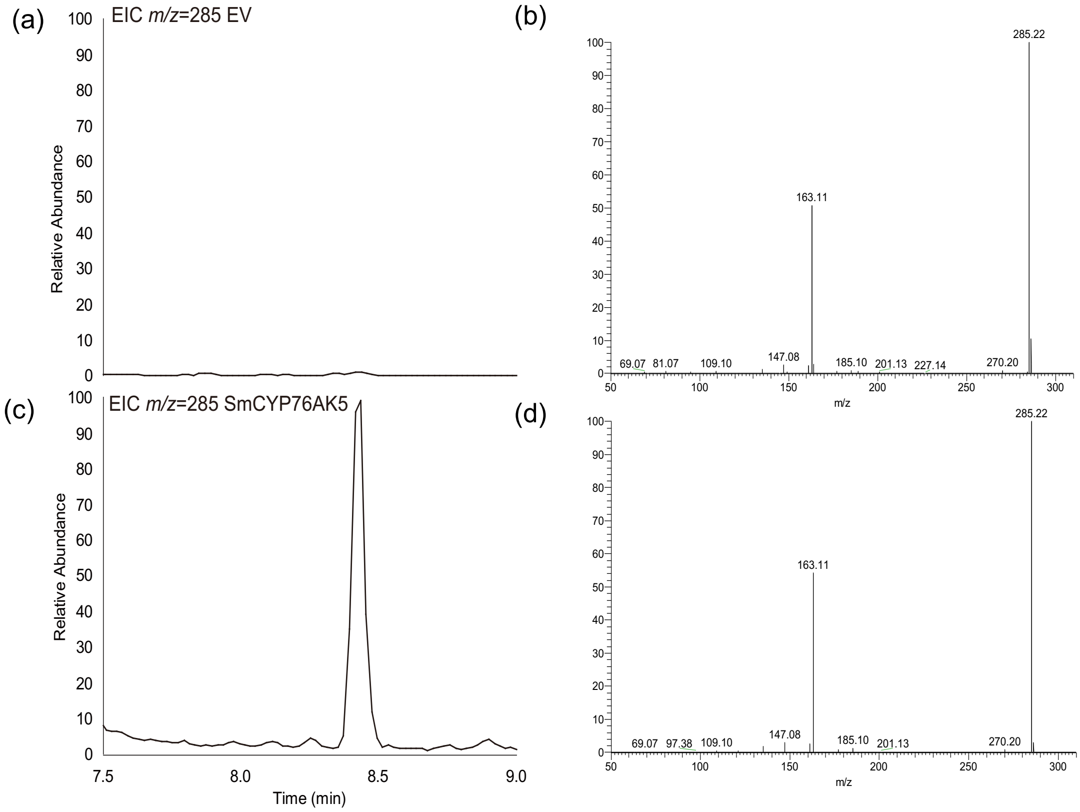

Fig. S16 Characterization of CYP76AK5 in yeast. (a) EIC of *S. cerevisiae* WAT11 harboring the empty vector. (c) EIC of S. cerevisiae WAT11 expressing SmCYP76AK5. (b) MS/MS fragmentation spectrum of the enzymatic product. (d) MS/MS spectrum of the corresponding authentic standard.

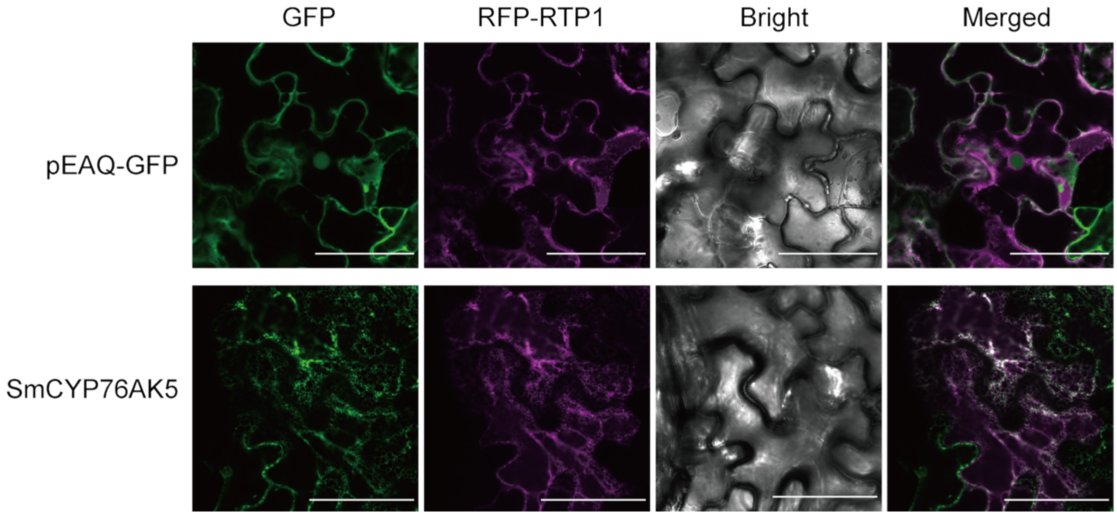

Fig. S17 Subcellular localization of SmCYP76AK5 fused at the C terminus to GFP in transiently transgenic *N. benthamiana* leaves imaged by confocal laser-scanning microscopy. RTP1, endoplasmic reticulum (ER)-localized protein marker. Scale bars, 50 μm.

(b)

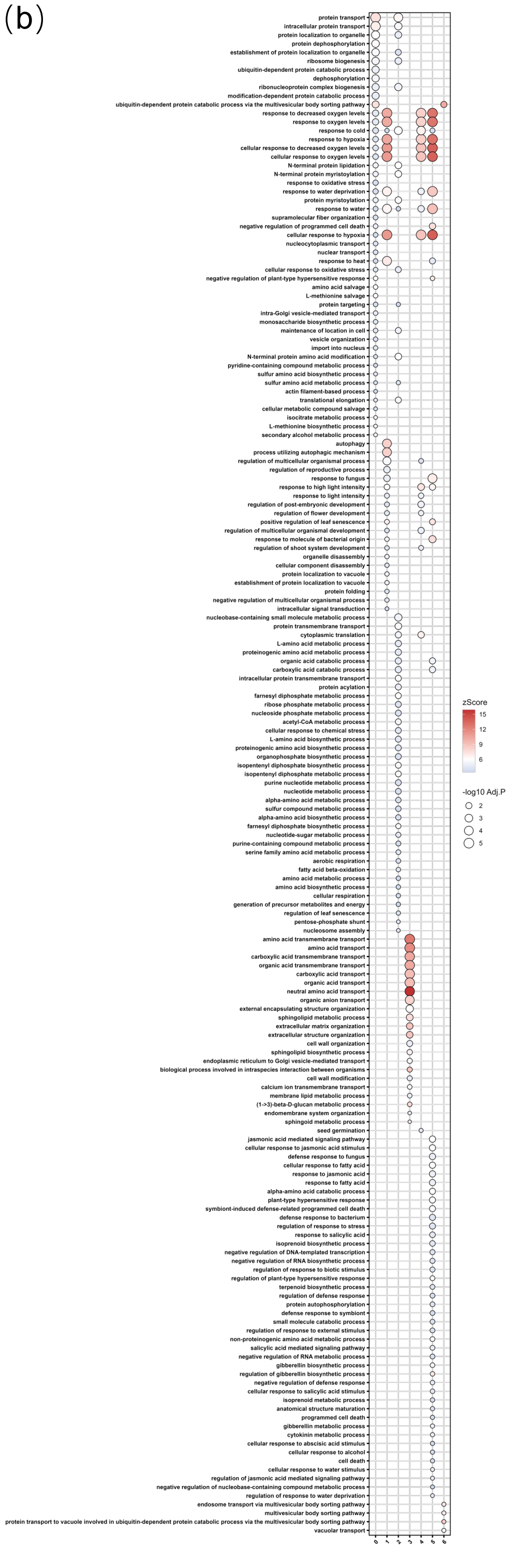

(a)

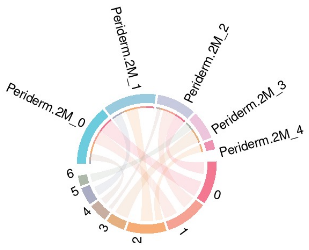

Fig. S18 Pathway enrichment analysis of 2M_3 (2M_C3). 2M_3 (2M_C3) is mainly associated with pathways 3 and 6. In the bubble plot, pathway 3 and pathway 6 represent the significantly enriched pathways for this module. The significance of enrichment is indicated by bubble size and color intensity, with larger and more intensely red bubbles indicating higher enrichment significance.

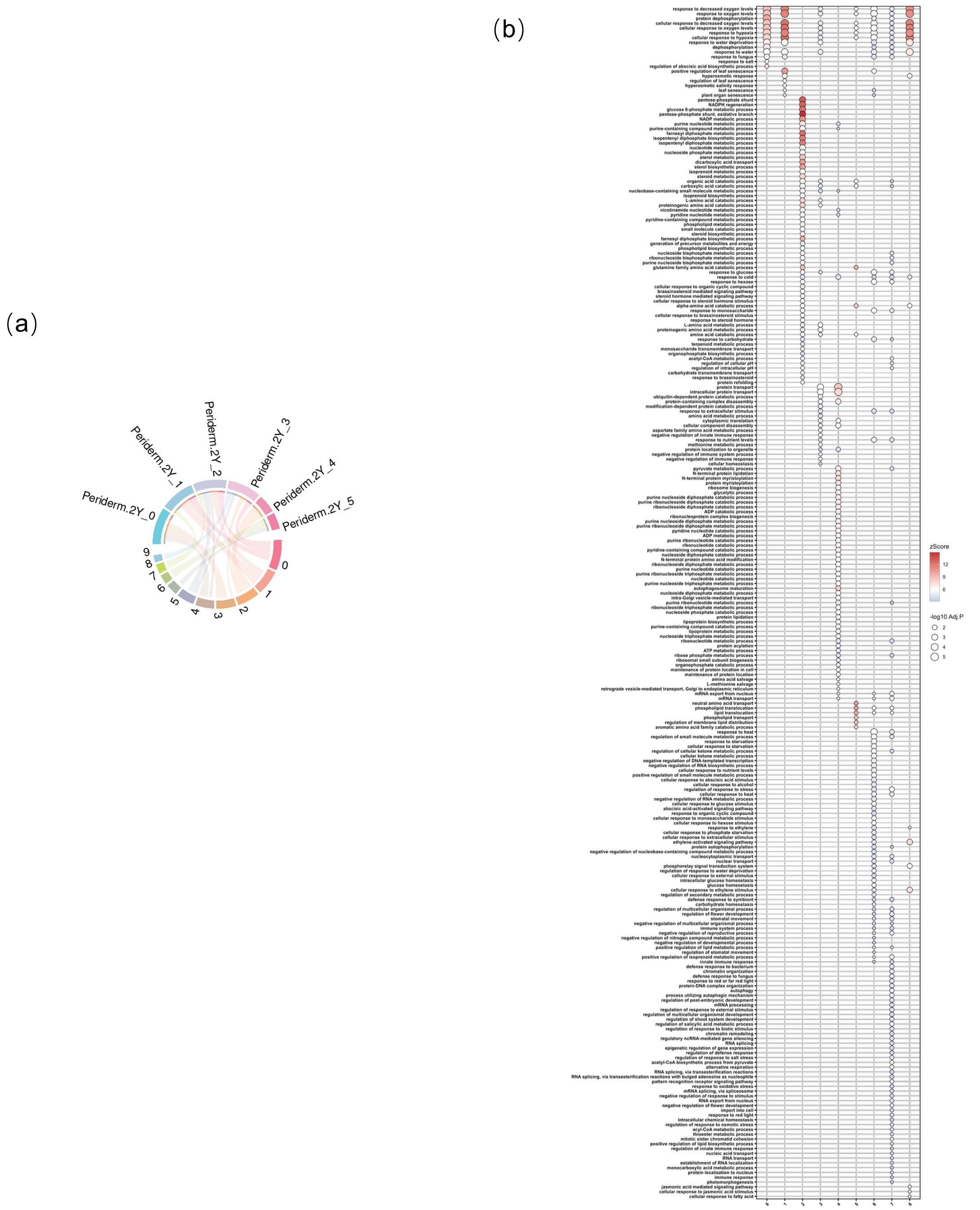

Fig. S19 Pathway enrichment analysis of 2Y_5 (2Y_C5). 2Y_5 (2Y_C5) is mainly associated with pathways 6 and 7. In the bubble plot, pathway 6 and pathway 7 represent the significantly enriched pathways for this module. The significance of enrichment is indicated by bubble size and color intensity, with larger and more intensely red bubbles indicating higher enrichment significance.

Reference

Chen J, Wang X, Ma A, Wang QE, Liu B, Li L, Xu D, Ma Q. 2022. Deep transfer learning of cancer drug responses by integrating bulk and single-cell RNA-seq data. *Nature Communications* **13**:6494.

Crow M, Paul A, Ballouz S, Huang ZJ, Gillis J. 2018. Characterizing the replicability of cell types defined by single cell RNA-sequencing data using MetaNeighbor. *Nature Communications* **9**:884.

Gretton A, Borgwardt KM, Rasch MJ, Schölkopf B, Smola A. 2012. A Kernel Two-Sample Test. *Journal of Machine Learning Research* **13**:723-773.

Khirirat S, Feyzmahdavian HR, Johansson M. 2017. Mini-batch gradient descent: faster convergence under data sparsity. *Proceedings of the 2017 IEEE 56th Annual Conference on Decision and Control (CDC)*: 2880-2887.
